# Strategic Fermi Level Engineering of Donor–Acceptor Self-Assembled Monolayer toward Ultrahigh Paired-Pulse Facilitations in Photosynaptic Transistors

**DOI:** 10.1021/jacs.5c11235

**Published:** 2025-09-15

**Authors:** Ya-Shuan Wu, Wei-Cheng Chen, Yi-Sa Lin, Cheng-Liang Liu, Yan-Cheng Lin, Wen-Chang Chen

**Affiliations:** a Department of Chemical Engineering, 33561 National Taiwan University, Taipei 10617, Taiwan; b Institute of Polymer Science and Engineering, National Taiwan University, Taipei 10607, Taiwan; c Department of Materials Science and Engineering, 33561 National Taiwan University, Taipei 10617, Taiwan; d Advanced Research Center of Green Materials Science and Technology, National Taiwan University, Taipei 10617, Taiwan; e Department of Chemical Engineering, 34912 National Cheng Kung University, Tainan 70101, Taiwan

## Abstract

Given the rapid advancement of artificial intelligence and the demand for low-power computing, photosynaptic transistors have emerged as promising candidates by integrating sensing, memory, and processing to mimic biological synapses. However, simultaneously realizing high paired-pulse facilitation (PPF) and low energy consumption in an artificial synapse, as biological ones, remains challenging. This property should be achieved by balancing the high carrier density while maintaining a low conductivity in the photoresponsive/charge-trapping electret; however, these prerequisites are mutually contradictory. To achieve this goal, a series of electron acceptors, 2,3,5,6-tetrafluoro-7,7,8,8-tetracyanoquinodimethane (F_4_TCNQ), tris­(pentafluorophenyl)­borane (BCF), and their Lewis-paired F_4_BCF, are introduced to the orderly anchored and segregated pyrene self-assembled monolayers for Fermi level modulation of the charge-trapping electret. The high electron deficiency of F_4_BCF induces strong donor–acceptor interaction with pyrene, lowering its Fermi level and stabilizing charges through charge transfer. This molecular design strategy yields a record-high PPF ratio of 293% and an ultralow energy consumption of 2.96 × 10^–19^ J, supporting low-power and multilevel memory characteristics analogous to those of biological synapses. Finally, the demonstrated image preprocessing highlights its potential for neuromorphic visual computing. This study highlights the effectiveness of Lewis-paired acceptor engineering as a powerful molecular strategy for modulating the Fermi level. By combining the segregated anchored SAM electret, a decent balance between high carrier density and low conductivity is achieved, thereby realizing the highest PPF ratio and the lowest energy consumption simultaneously among the reported systems. This outperformance underscores its potential for next-generation low-power optoelectronic neuromorphic devices.

## Graphical abstract

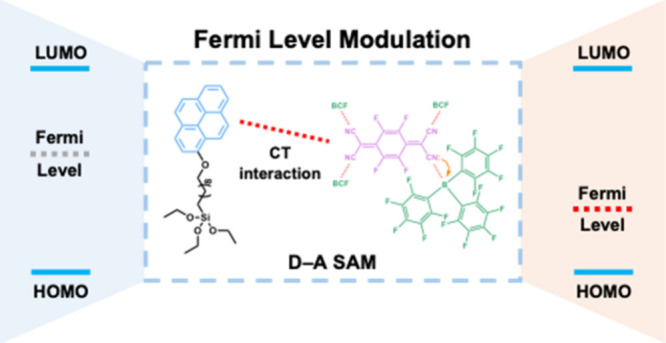

## Introduction

With the rapid development of artificial intelligence (AI) and the growing demand for high-speed and large-scale information processing, the limitations of the conventional von Neumann architecture in achieving highly parallel and energy-efficient operations similar to those of the human brain have become increasingly apparent. In conventional computing systems, the physical separation between memory and the processing unit results in substantial energy consumption due to the frequent and intensive data transfer.^1,2^ In contrast, the power consumption is as low as 20 W, and the energy consumption required for each synaptic event is estimated to be only 1–100 fJ in the human brain.^3,4^ This outstanding efficiency underscores the biological advantage of integrated memory and computation. In biological systems, synapses are crucial for transmitting information between neurons via electrical or chemical signals.^5^ Therefore, synaptic transistors have garnered significant attention in the current era of information-driven technological advancement. By integrating signal transmission, sensing, processing, and memory functions within a single architecture, synaptic transistors offer a promising solution to simultaneously reduce energy consumption and enhance processing efficiency, while enabling versatile functionality that realizes neuromorphic computing, emulating the capabilities of biological neural networks.

Building upon this foundation, it is worth noting that approximately 80% of the external stimuli perceived by humans is acquired through visual perception, with the retina functioning as a key biological interface for recognizing light signals and initiating initial neural processing.^6,7^ Accordingly, light-driven photosynaptic transistors are investigated to emulate the biological functions. Compared to conventionally electrically driven devices, optical stimulation offers unique advantages, including noncontact programming, low power consumption, reduced data latency, low crosstalk, and high bandwidth.^8,9^ The visual signal can also be directly transferred to an electrical signal efficiently using a photosynaptic transistor. The design of the charge-trapping layer is critical in developing a high-performance photosynaptic transistor. The commonly adopted materials can be broadly classified into three types: (i) inorganic materials,^10–12^ (ii) polymer electret,^13–15^ and (iii) hybrid systems.^16–18^ However, despite their functional advantages, the relatively large thickness of these materials restricts their compatibility with advanced high-density fabrication processes. This limitation poses significant challenges for developing highly integrated and miniaturized neuromorphic devices. Self-assembled monolayers (SAMs) have emerged as promising ultrathin interfacial materials for optoelectronic devices, addressing this issue. Their well-ordered and densely packed structures enable precise modulation of interfacial properties at the molecular scale.^19,20^ Besides their structural uniformity, SAMs can be fabricated through simple processes, offering excellent scalability and cost-effectiveness.^21^ Furthermore, the intrinsic surface passivation effect of SAMs effectively suppresses interfacial trap states and improves charge stability.^22,23^ These merits make SAMs compatible with high-density and miniaturized device architectures.

In recent years, donor–acceptor (D–A) interactions have been introduced to enhance the electronic properties of devices and further modulate energy levels and charge dynamics.^24–26^ This noncovalent interaction involves charge transfer (CT) between an electron-rich donor and electron-deficient acceptor units. Owing to their strong electron deficiency and ability to stabilize charge carriers, such acceptor molecules are usually employed to improve the electrical conductivity of organic semiconductors through the doping mechanisms.^27,28^ For instance, Shen et al. reported that thermally evaporated 2,3,5,6-tetrafluoro-7,7,8,8-tetracyanoquinodimethane (F_4_TCNQ) on an ultrathin conjugated polymer layer markedly enhanced its conductivity to 10^3^ S/cm, with a low sheet resistance of 10^3^ Ω/square. This improvement was attributed to the high hole mobility and effective CT induced by the dopant, enabling the fabrication of fully organic, transparent field-effect transistors with superior mobility and over 90% optical transmittance.^29^ Recently, Weng et al. demonstrated that doping with tris­(pentafluorophenyl)­borane (BCF) improved both carrier mobility and stretchability in polymer semiconductors, which resulted from dopant incorporation into side-chain regions, facilitated by increased lamellar spacing and reduced crystallinity.^30^ On the other hand, recent studies have demonstrated that charge transfer concepts can be effectively introduced into synaptic phototransistors to enhance device performance and functionality. For instance, a Rhodamine 6G/InSe hybrid architecture was shown to promote interfacial CT at the organic/inorganic interface, thereby boosting photocurrent generation and improving memory characteristics for secure optical encryption applications.^31^ Similarly, incorporating F_4_TCNQ into conjugated polymers such as PQT-12 and P3HT effectively modulated the electronic structure through effective CT interactions and extended the operational wavelength range of the devices into the near-infrared region.^32,33^ These advances underscore the potential of CT engineering in tailoring optoelectronic responses and optimizing synaptic behavior in phototransistors. Although conventional electron acceptors have been shown to boost the electrical performance of p-type semiconductors, the efficiency of CT is fundamentally constrained by the energy level alignment between the highest occupied molecular orbital (HOMO) level of donors and the lowest unoccupied molecular orbital (LUMO) level of acceptors. Therefore, to broaden the applicability of electron acceptors across diverse host materials, a higher electron deficiency (i.e., a lower-lying LUMO level) is required to ensure effective CT and further lead to a deeper Fermi level of hosts. One promising strategy involves Lewis-paired dopants, in which the Lewis acids are coordinated with cyano-functionalized molecules to strengthen the electron-withdrawing capability of the resulting complex.^34–36^ The bulky nature of these complexes also contributes to excellent electrical stability by suppressing dopant migration and minimizing structural reorganization under operation.^34^ While Lewis-paired strategies have demonstrated considerable success in enhancing the conductivity of organic semiconductors, their application in modulating the Fermi level of charge-trapping layers in synaptic transistors to improve charge trapping efficiency and stabilize charge retention has not yet been explored.

To realistically emulate the computational principles of biological synapses, achieving a high paired-pulse facilitation (PPF) ratio is crucial. PPF represents a hallmark of short-term synaptic plasticity, which underlies essential neural functions such as signal amplification, temporal learning, and sensory adaptation. A high PPF ratio reflects the device’s ability to respond more strongly to successive stimuli, mimicking the facilitation effect observed in biological neural circuits. In parallel, minimizing energy consumption per synaptic event is critical to replicating the brain’s exceptional energy efficiency. However, the concurrent realization of both high PPF and ultralow energy operation remains a significant challenge. Herein, a series of D–A SAMs (DA-SAMs) were designed to examine the effect of the Fermi level in the charge-trapping layer on the synaptic performance. A silane-based SAM incorporating a pyrene conjugated core (Py) was employed as the donor and coordinated with three distinct electron acceptors to form the DA-SAM systems with varying electron-withdrawing capabilities, which are F_4_TCNQ, BCF, and a Lewis-paired complex (F_4_BCF) formed by coordination between one F_4_TCNQ and four BCF molecules, respectively. The CT interactions and coordination behavior between Py and the electron acceptors were systematically analyzed using X-ray photoelectron (XPS) and ultraviolet photoelectron spectroscopy (UPS). The result shows that the Lewis-paired F_4_BCF, which possesses the highest electron deficiency, induced the most pronounced downshift of the Fermi level in the charge-trapping layer toward the HOMO level of Py. This electronic modulation resulted in the strongest charge-trapping capability and significantly improved device stability. The F_4_BCF-based system achieved the highest PPF (293%) reported to date and exhibited ultralow energy consumption of 2.96 × 10^–19^ J. These findings validate the simultaneous realization of high PPF and ultralow energy consumption through DA-SAM engineering. This approach outperforms conventional spin-coated devices, highlighting the effectiveness of combining SAM design with strong electron acceptors in achieving efficient synaptic behavior. It establishes a novel application pathway for SAM-based molecular engineering in synaptic devices.

## Results and Discussion

### Conceptual Framework for Energy-Level Design in the DA-SAM System

In a p-type phototransistor device, charge storage typically relies on the accumulation of electrons in the charge-trapping layer. The charge-trapping layer must effectively stabilize the captured electrons to achieve stable and long-lived charge retention. From an energetic perspective, electrons in lower-lying energy levels are less susceptible to thermal or field-induced detrapping. Therefore, a higher electron deficiency in the trapping material is required to create deeper energy states for efficient electron stabilization. One promising strategy to modulate the energy level involves incorporating electron acceptors, which allow for precise tuning of the Fermi level within the charge-trapping layer.^37,38^ The bottom gate/top contact (BG/TC) device is presented in Figure 1a. The Py SAM was grown on a silicon wafer with an oxide dielectric layer. This was followed by coordination with the electron acceptors through immersion in acceptor solutions to form the CT complexes. To prevent unintended doping of the channel layer, a thin hole transporting layer (HTL), poly­(9-vinylcarbazole) (PVK), was spin-coated before the thermal evaporation of dinaphthothienothiophene (DNTT) and Au, which were deposited sequentially as the semiconducting layer and the electrodes, respectively. Figure 1b shows the chemical structures of all the studied materials. The BCF system interacts with traces of water molecules to form the Bronsted acid.^30^ It is worth noting that F_4_BCF is a Lewis acid–base complex composed of F_4_TCNQ and four equivalents of BCF. The interaction between the electron-donating cyano groups in F_4_TCNQ and the electron-deficient boron centers in BCF forms a stronger electron-withdrawing system. The enhanced electron deficiency results in a pronounced downshift of the LUMO energy level, as provided in Figure 1d, and the LUMO levels of the studied electron acceptors are – 5.20, – 5.30, and – 5.93 eV for F_4_TCNQ, BCF, and F_4_BCF, respectively.^34,39–41^ Since the work function (WF) reflects the energy barrier for electron removal, an increase in WF indicates a greater energetic resistance to electron loss. Upon the formation of D–A interactions between Py and the acceptors, the Fermi level of the charge-trapping layer was lowered, thereby raising the WF. This deeper energy level contributed to improved CT efficiency (Figure 1e) and enhanced the device’s current stability (Figure 1e), especially in the case of F_4_BCF. The correlation of the D–A interactions with the optoelectronic characteristics will be further corroborated.

**Figure 1:**
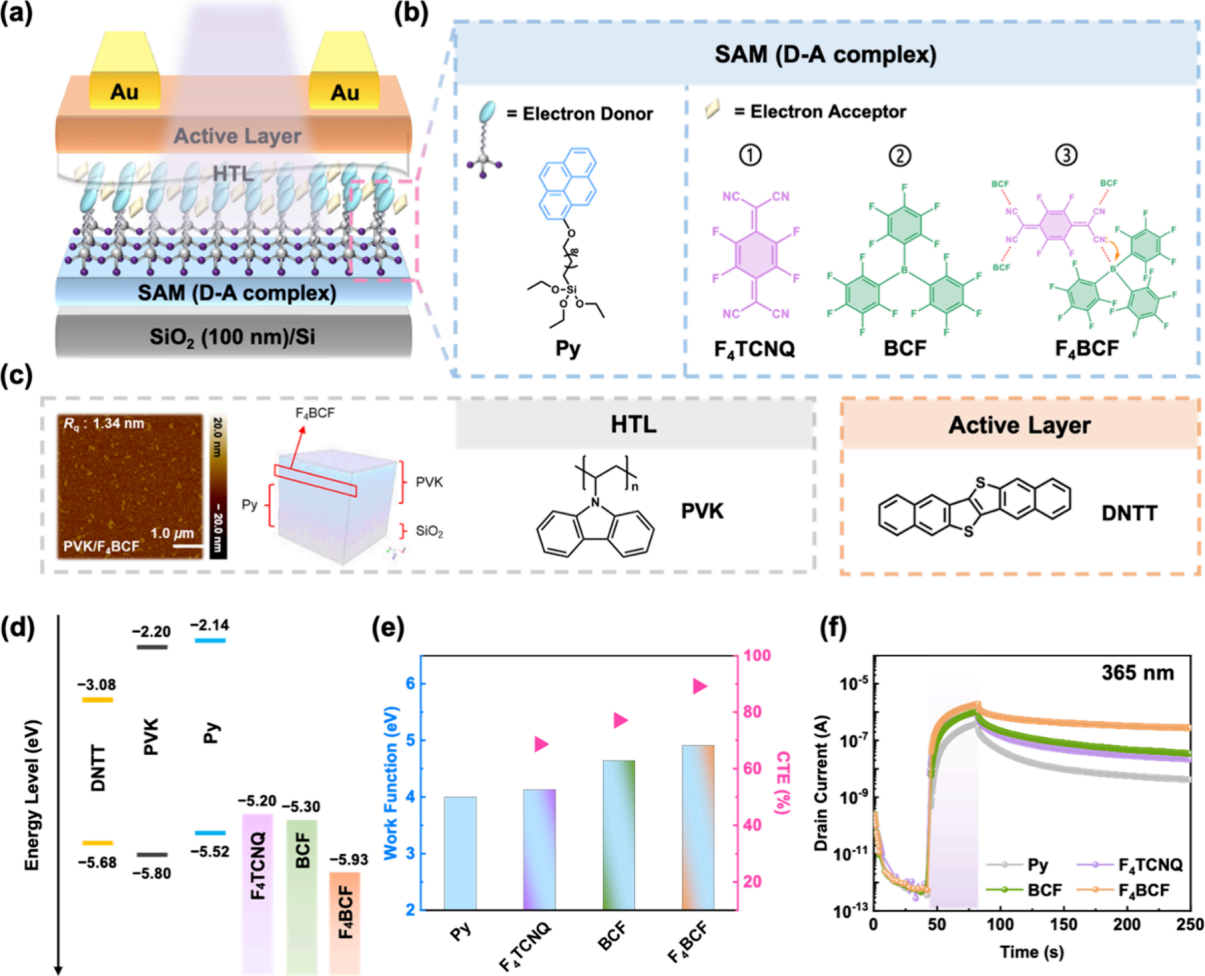
(a) Device architecture of the DA-SAM-based photosynaptic transistor. (b) Chemical structures of the constituent materials: a donor-type SAM, the electron acceptors, the hole-transporting layer of PVK, and the channel of DNTT. (c) AFM height image (left) and ToF-SIMS 3-D mappings (right) of PVK/F_4_BCF structure. (d) Energy levels of the materials studied. (e) Relationship between work functions and charge transfer efficiencies of the D–A systems. (f) Transient characteristics of the DA-SAM-based phototransistors under 365 nm-light illumination for 40 s at *V*_d_ = – 50 V.

### Characterization of the Lewis-Paired Acceptor and the CT Complexes

After elucidating the research concept, the detailed analyses of the designed system were conducted. First, the formation of F_4_BCF was characterized using water contact angle measurement (Figure S1). The result shows that the water contact angle of F_4_BCF (84.6°) is similar to that of BCF (84.8°), rather than to an average of F_4_TCNQ and BCF, indicating that the F_4_TCNQ core is surrounded by BCF molecules in the F_4_BCF complex. This suggests that BCF moieties dominate the molecular arrangement at the surface of F_4_BCF. In addition, XPS analysis was conducted to reconfirm the Lewis acid–base coordination. In the B-1s spectrum, an additional deconvolution signal at 193.9 eV was observed in F_4_BCF compared to BCF, which can be attributed to the interaction between the CN group and the boron center (Figure 2 **a,b**).^42^ To further investigate the coordination behavior between F_4_TCNQ and BCF, ^19^F NMR spectroscopy was performed with different F_4_TCNQ:BCF ratios (Figure S2). The fluorine signals of BCF exhibit a noticeable upfield shift upon coordination, which can be attributed to the increased electron density surrounding the boron centers. In addition, F_4_TCNQ shows electron redistribution after coordination, resulting in the appearance of two distinguishable peaks. Based on the integration of the F_4_TCNQ and BCF signals, the relative ratio between the components can be inferred. These results evidence the successful formation of the Lewis-paired F_4_BCF complex, which was subsequently incorporated into DA-SAM systems to investigate its impact on Fermi level modulation and synaptic device performance.

**Figure 2:**
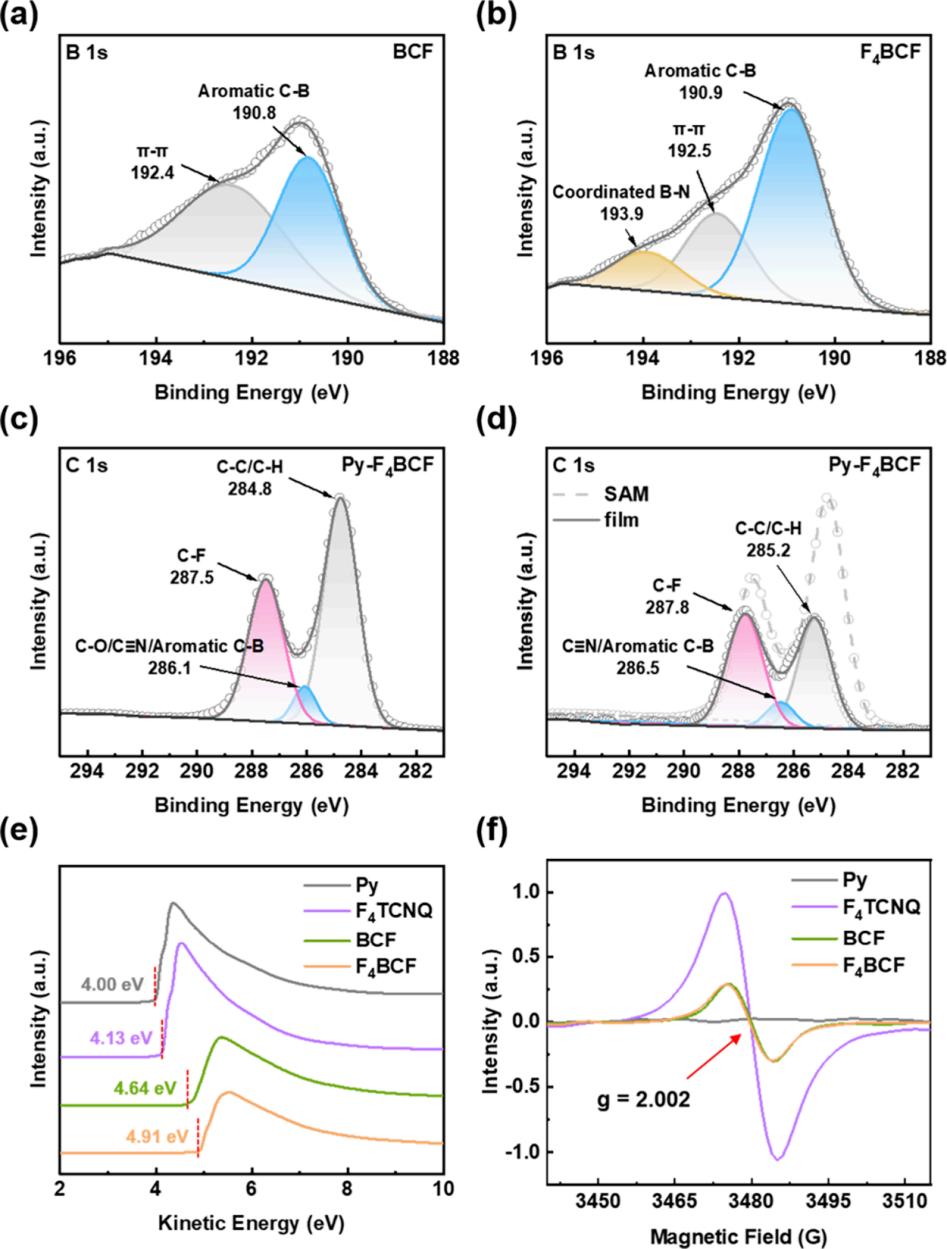
(a–d) XPS peak deconvolutions of the (a, b) B 1s and (c, d) C 1s bands for (a) pure BCF, (b) pure F_4_BCF, (c) Py–F_4_BCF complex, and (d) Py–F_4_BCF complexes with different fabrication processes to compare the SAM and thin-film systems. (e) UPS spectra of the D–A systems for the WF evaluations and the charge transfer levels. (f) EPR spectra of the D–A solutions with an acceptor concentration of 10 mol % to evaluate the contents of unpaired electrons during the charge transfer.

Next, C-1s and F-1s spectra were further analyzed to verify the D–A interaction between Py and the electron acceptors. As presented in Figures 2c and S3, compared to the pristine Py SAM, the peaks emerge at higher binding energy, which are 287.3 to 287.5 eV for F_4_TCNQ to F_4_BCF, respectively, and primarily originate from the intrinsic C–F bonding within the acceptor molecules. Upon coordination with the Py SAM, these peaks shift slightly toward lower binding energy, indicating electron transfer from Py to the acceptors and thus confirming the D–A interaction at the interface (Figures S3a **–f**). Density functional theory (DFT) calculations indicate that hydrogen bonding is unlikely due to the relatively long F···H distance (∼3.74 Å) between the pyrene and F_4_TCNQ molecules, which is also consistent with the ^1^H NMR spectra showing no significant chemical shift (Figures S3g,h). A slight peak broadening in the NMR spectra may be attributed to a small fraction of uncoordinated acceptors or CT-induced molecular aggregation. On the other hand, the F-1s spectra of the electron acceptors and the DA-SAMs are demonstrated in Figure S4. Notably, the peaks in all three systems exhibit downshifts in binding energy, which alter from 688.8–688.2 eV for the electron acceptors to 687.6 eV for the DA-SAMs. To highlight the structural advantages of the SAM-based architecture, XPS measurement on a spin-coated pyrene–acceptor blend film was also performed for comparison. As shown in Figure 2d, the DA-SAMs exhibit significantly stronger signal intensity than their spin-coated counterpart. This enhancement can be attributed to the densely packed and well-ordered arrangement of the SAMs, which promotes intensified D–A interactions and a more substantial alteration of the electronic environment at the interface. The result indicates that the introduction of acceptors affects the electronic structures of the complexes. In this regard, the D–A interaction evidenced in XPS data forms the basis for the modulation of the Fermi level and charge-trapping behavior in device operation. To further investigate this modulation effect, UPS was utilized to understand the WF variations across the CT systems.^43^ The corresponding equations for calculating the WFs are shown below, and the results are presented in Figure 2 **e:**

$$E_{\mathrm{kin}}=h\nu -E_{B}$$ 1

$$\Phi =h\nu -E_{\mathrm{cutoff}}$$ 2

where *E*_kin_ represents the kinetic energy, *hv* = 21.2 eV is the photon energy of the excitation source, *E*_B_ stands for the binding energy measured by the UPS, Φ is the WF, and *E*_cutoff_ represents the secondary electron cutoff energy determined from the onset of the high *E*_B_ region. The WFs for Py, F_4_TCNQ, BCF, and F_4_BCF are 4.00, 4.13, 4.64, and 4.91 eV, respectively. The higher value obtained in the F_4_BCF system can be ascribed to the more substantial electron deficiency provided by the Lewis acid–base interaction, leading to a more electron-deficient property and an increased energy barrier for electron loss. In addition, electron paramagnetic resonance (EPR) was carried out to investigate the presence of radical species and further elucidate the CT behavior within the D–A complexes. As shown in Figure 2f, no EPR signal was detected in Py, indicating the absence of single-spin electrons. In contrast, after coordination with the electron acceptors, a signal centered at a g-value of 2.002 was observed, suggesting the generation of unpaired electrons associated with D–A interaction.^44,45^ Interestingly, the EPR intensities presented in the BCF and F_4_BCF systems are lower than those of the F_4_TCNQ counterpart. The origin of this phenomenon stems from spin pairing within tightly bound CT complexes, making them less detectable by EPR.^46,47^ This indicates that the interaction between Py and the acceptors facilitates substantial CT and unpaired electron stabilization.

### Optical Characterization and Photophysics of the CT Systems

After investigating the interactions between donors and acceptors, the steady-state and transient optical characteristics were studied, as light plays a fundamental role in the operation of optoelectronic devices. First, the solution-state UV–vis absorption spectra with an acceptor concentration ranging from 0 to 50 mol %, along with those of the pure acceptors, are provided in Figures 3a and S5a,b. As can be seen, the absorption band of pure Py is around 300–375 nm. In the case of F_4_TCNQ, since the pure F_4_TCNQ exhibits strong absorption in the 300–600 nm range, the absorbance observed at 350–415 nm increases with rising concentration, reflecting its intrinsic spectral contribution. Notably, in BCF and F_4_BCF systems, although the pure acceptors present negligible absorption in the UV–vis region, the absorption band of Py shows a bathochromic shift upon complex formation. This result may originate from electronic interactions analogous to those previously reported for polaron formation in doped polymer systems.^48,49^ In the D–A complexes, the red-shift features can be associated with charge redistribution or structural reorganization within the donor upon coordination, even in the absence of distinct acceptor absorption.

**Figure 3:**
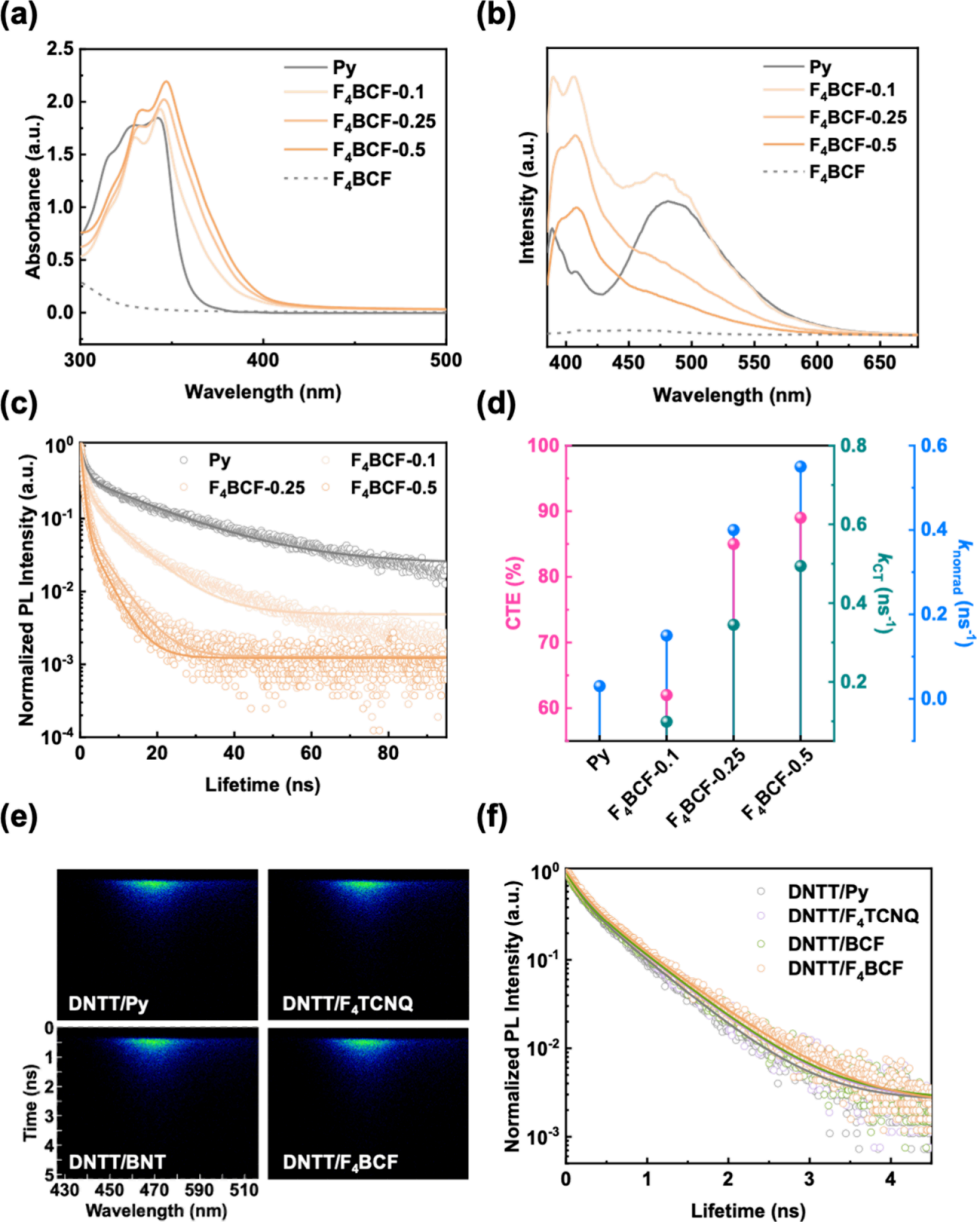
(a) UV–vis absorption spectra, (b) steady-state PL emission spectra, (c) 1D TR-PL decay profiles, and (d) nonradiative parameters including CTE, *k*_CT_, and *k*_nonrad_ of the Py–F_4_BCF solutions with an acceptor concentration of 0–50 mol %. TR-PL (e) 2D patterns and (f) 1D decay profiles of DNTT deposited on different DA-SAMs. Note that the λ_ex_s are (b, c) 365 and (e, f) 375 nm.

Furthermore, the solution-state photoluminescence (PL) was measured, as shown in Figures 3b and S5c,d. In the PL spectra, the emission range for pure Py spans from 385 to 650 nm. Since the favorable energy level alignment, the strong D–A interaction between Py and F_4_BCF leads to an enhanced emission in the shorter-wavelength region, which indicates that the molecular packing of Py was perturbed by the introduction of F_4_BCF, promoting amorphous-phase emission associated with high-energy transition. In addition, the PL intensity decreases with increasing F_4_BCF concentration, which suggests that the strengthened CT interaction facilitates the nonradiative recombination process. The F_4_TCNQ system exhibits a similarly decreasing trend in PL intensity due to the CT-induced quenching. Therefore, the result underscores that the CT interaction with electron-deficient acceptors induces notable electronic modulation of the donor, as reflected in the altered optical absorption behavior. Interestingly, the BCF system presents an increase in PL intensity with increasing acceptor concentrations. This can be attributed to the structural effect, where BCF coordination results in reduced molecular aggregation of Py. The corresponding emission images of the CT solutions are demonstrated in Figure S6.

In addition to the steady-state optical analyses, time-resolved photoluminescence (TR-PL) was further explored to evaluate the transient photophysics. The 2-D TR-PL patterns of the D–A complexes are shown in Figure S7, and the corresponding 1-D decay profiles are presented in Figure 3c and S5e,f. The 1-D decay profiles were fitted using a biexponential function:

$$I\left(t\right)=I_{0}+A_{1}\exp\left(-t/\tau_{1}\right)+A_{2}\exp\left(-t/\tau_{2}\right)$$ 3

where *I* represents the time-dependent PL intensity, *t* stands for the fluorescence decay time, *A*_1_ and *A*_2_ are the scaling constants, and τ_1_ and τ_2_ are the radiative and nonradiative components of PL decay lifetime. Then, the average exciton lifetime (τ_avg_) was calculated as shown below:

$$\tau_{\mathrm{avg}}=\left(A_{1}{\tau_{1}}^{2}+A_{2}{\tau_{2}}^{2}\right)/\left(A_{1}\tau_{1}+A_{2}\tau_{2}\right)$$ 4

The optical parameters of the CT complexes are summarized in Table S1. The τ_avg_ for Py is 16.57 ns, and the lifetimes reduce after the formation of the CT complexes. The τ_avg_ decreases from 12.60–5.20, 4.53–3.80, and 6.29–1.80 ns for the F_4_TCNQ, BCF, and F_4_BCF systems, respectively, as the acceptor concentration increases from 10 to 50 mol %. This result reflects an elevated proportion of nonradiative recombination, suggesting a stronger D–A interaction, which facilitates exciton dissociation or quenching via CT mechanisms. Therefore, the charge transfer efficiency (CTE) and the charge transfer rate (*k*_CT_) were further calculated using the following equations:

$$\mathrm{CTE}=\left(\tau_{\mathrm{Py}}-\tau_{\mathrm{CT}\;\mathrm{copmlex}}\right)/\tau_{\mathrm{Py}}\times 100\%$$ 5

$$1/\tau_{\mathrm{CT}\;\mathrm{copmlex}}=1/\tau_{\mathrm{CT}\;\mathrm{copmlex}}+1/\tau_{\mathrm{CT}};k_{\mathrm{CT}}=1/\tau_{\mathrm{CT}}$$ 6

Notably, the CTE (%) and *k*_CT_ (ns^–1^) rise from (69, 0.132), (77, 0.203), and (89, 0.494) for F_4_TCNQ, BCF, and F_4_BCF systems, respectively, with an acceptor concentration of 50 mol %. The result shows that the F_4_BCF system, which possesses the strongest electron-withdrawing capability, demonstrates the most pronounced CT interaction. This is consistent with the UPS results, where the Fermi level of the trapping layer shifts closer to the HOMO level of the donor, facilitating more effective electron trapping and stable charge retention. Along with the photoluminescence quantum yield (PLQY) measurement, the radiative (*k*_rad_) and nonradiative (*k*_nonrad_) recombination rates can be calculated as follows:

$$1/\tau_{\mathrm{avg}}=k_{\mathrm{rad}}+k_{\mathrm{nonrad}}$$ 7

$$\mathrm{PLQY}=k_{\mathrm{rad}}/\left(k_{\mathrm{rad}}+k_{\mathrm{nonrad}}\right)$$ 8

As can be seen, in all cases, the dominance of *k*_nonrad_ over *k*_rad_ highlights the CT dynamics in the D–A systems, which directly influence the charge trapping and synaptic behavior of the photosynaptic transistor.

In addition to the charge-trapping layer, the photoresponse of the semiconducting layer also plays a crucial role in a transistor device. Therefore, the transient optical properties of DNTT layered on different DA-SAMs were examined by the TR-PL analyses. The 2-D TR-PL patterns and the corresponding 1-D decay profiles are provided in Figure 3 **e,f**, respectively, and the calculated parameters are listed in Table S2. It is worth noting that the DNTT deposited onto F_4_BCF exhibits the longest τ_avg_ and the lowest *k*_nonrad_, which indicates that the CT interaction between Py/F_4_BCF effectively passivates the defects in DNTT, thereby reducing energy loss and improving the photoresponse.

### Device Performance of the Phototransistors Based on the DA-SAMs

Considering the critical impact of interfacial morphology on device performance, atomic force microscopy (AFM) was conducted to analyze the surface morphology of PVK films spin-coated on different DA-SAMs before fabricating the devices. The height images were presented in Figures 1c and S8, and the result shows their low surface roughness of 1.03, 0.98, 1.14, and 1.34 nm for Py, F_4_TCNQ, BCF, and F_4_BCF, respectively. Thus, in this device architecture, the implementation of PVK offers three interfacial functions: (i) improving morphological uniformity, (ii) preventing doping of the semiconducting layer, and (iii) serving as an HTL for hole transport facilitation. In addition, 3-D mappings of time-of-flight secondary ion mass spectrometry (ToF-SIMS) were used to identify the spatial distribution of each layer within the device (Figures 1c and S9,10). The 1-D depth profiles in Figure S9 demonstrate the formation of an ultrathin Py monolayer (<3 nm), and the acceptor signals are found to overlap with the surface of the Py layer, indicating the D–A interaction between the conjugated core and the acceptor through coordination. After spin-coating PVK, the results are shown in Figure S10. Overall, these findings confirm a well-defined device architecture composed of a monolayer SAM, molecular-level D–A coordination, and a PVK interlayer functioning as a protective layer. Furthermore, the morphology of the channel layer has an essential impact on charge transport, and a uniform molecular arrangement within the active layer can facilitate carrier mobility and enhance device performance. As a result, AFM measurements were conducted on DNTT films deposited atop PVK-modified SAMs, as provided in Figure S11. As can be seen, the DNTT films exhibit comparable and uniform surface morphology across all four samples, regardless of the underlying DA-SAM. This morphological similarity further confirms the function of PVK as an effective interfacial modifier that homogenizes the surface properties, which is beneficial for ensuring stable charge transport and reliable synaptic response.

### Memory Behavior of the Phototransistors

Multiple factors, including interfacial morphology, energy level alignment, and the intrinsic behavior of the channel, can determine the electrical performance of a transistor device. Based on the previous results, the effect of CT on Fermi level modulation and its influence on the device charge-trapping capability are investigated. First, the dual sweep transfer curves were measured with a gate voltage (*V*_g_) sweeping forward and backward between 20 and – 60 V at a drain voltage (*V*_d_) of – 50 V to verify the hysteresis, as presented in Figure S12. It is worth noting that the memory window only appears in the presence of SAMs, confirming that the observed hysteresis originates from the charge-trapping layer. According to the energy level alignment, the donor strength toward F_4_BCF decreases in the order Py, DNTT, and PVK. Thus, F_4_BCF mainly coordinates with the Py SAM, and the spin-coated PVK layer does not interfere with this interaction. UPS measurements further confirm the negligible effect of F_4_BCF on PVK (Figure S12g), while control experiments show that blending F_4_BCF into PVK leads to DNTT doping and a high OFF current, which is absent in the devices (Figures S12h **,i**). Next, the transfer characteristics were conducted at *V*_d_ = – 50 V with a *V*_g_ ranging from 20 to – 60 V (Figures 4a and S13). When a negative bias (*V*_g_ = – 60 V) was applied for 1s, all systems present a negative shift in the transfer curves, corresponding to the OFF state. Subsequently, the devices were illuminated using 365 nm light (3.20 mW/cm^2^) for 40 s to induce the ON state transition. The result indicates that, compared to the Py system, in which the transfer curve returns to its initial state after programming, SAMs with D–A interactions exhibit a noticeable positive shift and present a bistable memory behavior, especially in the F_4_BCF system. The threshold voltages and the memory windows (*V*t_h,write_, *V*_th,erase_, Δ*V*_th_) are (−24.2, – 0.1, 24.1) V, (−23.0, 1.0, 24.0) V, (−21.0, 1.1, 22.1) V, and (−19.2, 2.6, 21.8) V for Py, F_4_TCNQ, BCF, and F_4_BCF, respectively. This can be ascribed to the enhanced electron deficiency of the charge-trapping layer, regulated by the acceptor-induced Fermi level modulation, which enables adequate storage of excess photogenerated electrons. Additionally, the device stability was characterized by the transient curves. As shown in Figure 1f, incorporating electron acceptors significantly enhances the current stability after light illumination (365 nm; 3.20 mW/cm^2^; 40 s) at *V*_d_ = – 50 V. Among all the systems, F_4_BCF shows the highest *I*_ON_/*I*_OFF_ of ∼ 10^6^, indicating the superior charge-trapping capability with the Lewis-paired complex. These findings highlight the significance of Fermi level tuning in enhancing the electrical performance and photoresponse of the optoelectronic device.

**Figure 4:**
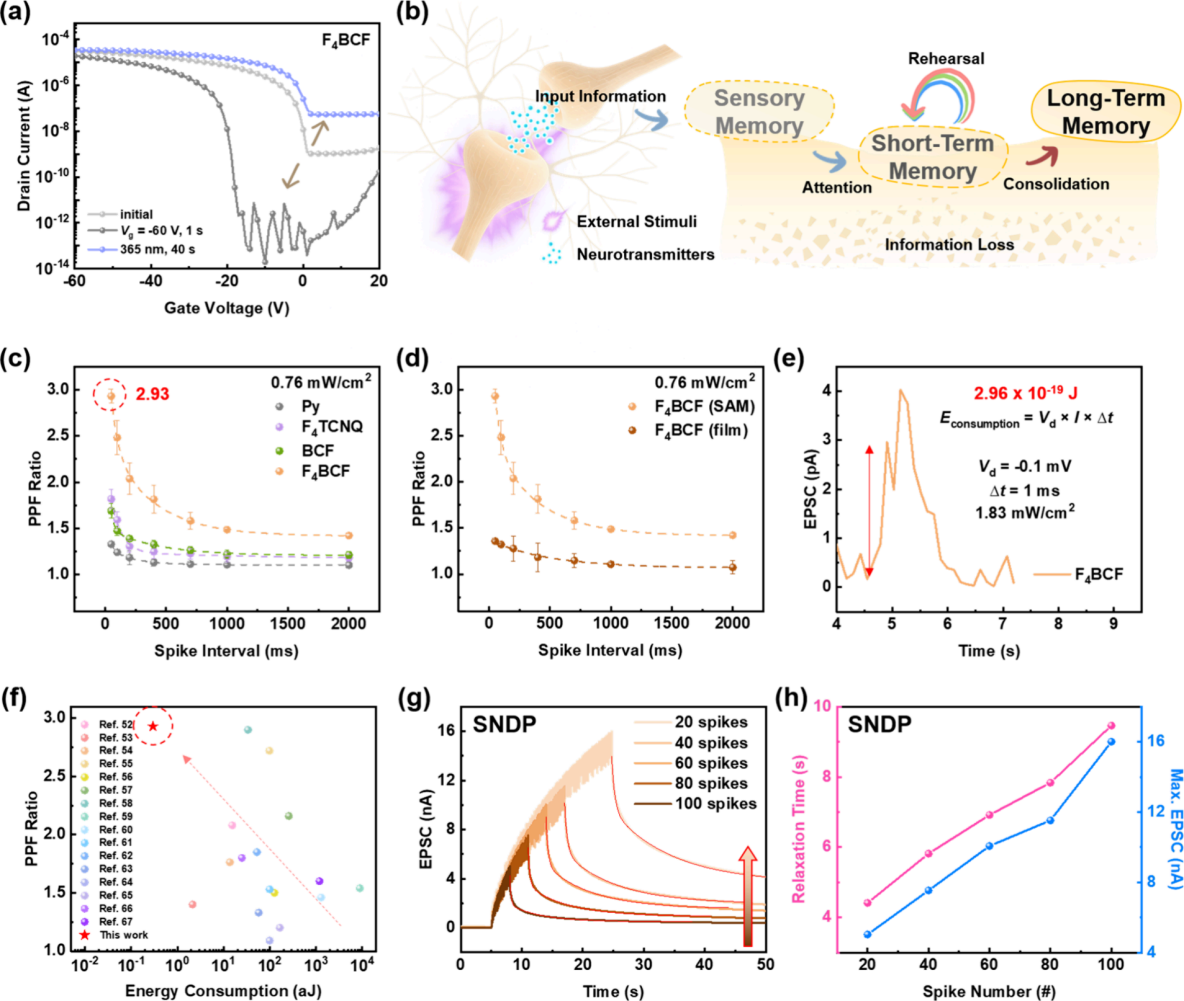
(a) Transfer characteristics of F_4_BCF-based device under the illumination of 365 nm light (3.20 mW/cm^2^) at *V*_d_ = −50 V. (b) Schematic diagram of the multistore memory model in the human brain. (c, d) Relationship between PPF ratios and spike intervals of (c) DA-SAM-based phototransistors and (d) spin-coated control device with 365 nm-light illumination (0.76 mW/cm^2^; spike width = 100 ms). (e) *E*_consumption_ per synaptic event under 365 nm light illumination (1.83 mW/cm^2^; spike width = 50 ms) at *V*_d_ = −0.1 mV. (f) Summary of the PPF ratios and *E*_consumption_s for the reported synaptic transistors: the device in this study simultaneously presents the record-high PPF ratio and record-low *E*_consumption_. (g) STM–LTM transitions and (h) relationship between relaxation times and maximum EPSCs for SNDP of the F_4_BCF device under 365 nm-light illumination at *V*_d_ = −1 V.

### Synaptic Characteristics and Human Learning Emulation

With the improved performance of DA-SAMs, the realization of multilevel storage remains an essential requirement for mimicking the behavior of the human brain. In a biological neural system, incoming information is transmitted between neurons through neurotransmitters upon receiving external stimuli. The multistore memory model in the human brain is depicted in Figure 4b.^50,51^ The input information is initially captured in sensory memory, then transferred into short-term memory (STM) through selective attention, and ultimately undergoes consolidation into long-term memory (LTM) for durable retention. To evaluate the synaptic plasticity of the devices, a series of synaptic performances were investigated to mimic critical biological functions. First, a typical indicator of STM in a photosynaptic device, PPF, is defined as the excitatory postsynaptic current (EPSC) ratio of two consecutive spikes (Figure S14), which the following equation can calculate:

$$\mathrm{PPF}=A_{2}/A_{1}\times 100\%$$ 9

where *A*_1_ and *A*_2_ are the first and second EPSCs, respectively. As can be seen in Figure 4c, the PPF ratios obtained at *V*_d_ = −1 V decay exponentially with increasing spike interval, which resembles biological synaptic behavior, in which closely spaced stimuli are more likely to induce effective memory formation. Furthermore, the enhanced charge stabilization provided by the acceptors facilitates more substantial short-term plasticity, making the D–A interaction beneficial for improving the PPF index. Notably, in the F_4_BCF system, where the Fermi level of Py SAM is substantially lowered through strong Lewis acid–base coordination, the device reaches an unprecedented PPF of 293%. As a result, the record-high PPF emphasizes the exceptional potential of energy level engineering in synaptic device optimization. To evaluate the superiority of SAM over conventional film deposition, a non-SAM-based control device was prepared by spin-coating a blended solution of pyrene and F_4_BCF. The PVK layer was then transferred atop the film for device fabrication (Figure S15). As provided in Figure 4d, despite the presence of D–A interactions, the control thin-film device presents a limited PPF ratio of 136%, underscoring the essential role of molecular ordering provided by the SAM structure. In addition, the energy consumption (*E*_consumption_) per synaptic event can be estimated as follows:

$$E_{\mathrm{consumption}}=V_{d}\times I_{\mathrm{peak}}\times \Delta t$$ 10

where *I*_peak_ represents the maximum EPSC, and Δ*t* is the spike width. Owing to the outstanding electron deficiency of the F_4_BCF-based charge-trapping layer, the device exhibits a pronounced photoresponse, which allows it to be triggered by extremely weak optical stimuli. Remarkably, an ultralow energy consumption of 2.96 × 10^–19^ J was achieved with a short light pulse (365 nm; 1.83 mW/cm^2^; 1 ms) at a low *V*_d_ of – 0.1 V, as shown in Figure 4e, which is several orders of magnitude lower than the energy required for a single synaptic event in biological systems. As summarized in Figure 4f, compared to the previous reported approaches, the design strategy combining SAMs with D–A interactions offers significant potential for application. This achievement is simultaneously the highest PPF and the lowest energy consumption reported in the literature.^52–67^ The synergistic effect of surface passivation by well-ordered SAMs and the charge stabilization provided by CT interactions boosts the synaptic device performance.

Next, the STM–LTM transition was demonstrated by manipulating the spike number (SNDP; 20–100 spikes), spike width (STDP; 150–350 ms), and spike intensity (SIDP; 0.40–1.30 mW/cm^2^) at *V*_d_ = −1 V based on the F_4_BCF system (Figures 4g and S16a,b). To quantitatively describe the memory behavior under different input conditions, the forgetting curves were fitted using modified Ebbinghaus’ psychological forgetting curve,^68,69^ as shown below:

$$P=\Delta G\left(t\right)/\Delta G_{\max}=B_{\mathrm{STM}}\exp\left[-{\left(t/\tau_{\mathrm{STM}}\right)}^{\beta }\right]+B_{\mathrm{LTM}}\exp\left[-{\left(t/\tau_{\mathrm{LTM}}\right)}^{\beta }\right]$$ 11

where *P* denotes the recall probability, Δ*G*(*t*) = *G*(*t*) – *G*_0_ is the conductance chance at time *t*, Δ*G*_max_ = *G*_max_ – *G*_0_ stands for the maximum conductance change relative to the initial state, τ_STM_ and τ_LTM_ represent the relaxation times corresponding to STM and LTM, and β is the stretching exponent (0 < β < 1), respectively. As shown in Figure 4h, when the spike number increases from 20 to 100, the maximum EPSC rises from 5 to 16 nA, and the relaxation time extends from 4.4 to 9.5s. Similar trends were observed in STDP and SIDP tests, where prolonged illumination or increased light intensity result in stronger synaptic responses and slower decay rates (Figure S16c,d). Finally, the relearning–forgetting–relearning process was demonstrated in Figure S17. The device was initially illuminated with 25 spikes (365 nm; 0.76 mW/cm^2^; spike width = 100 ms) to improve the photoconductivity for the learning process, which faded over 15 s as the forgetting process. Remarkably, the same response was recovered with only 4 spikes, showcasing the memory retention analogous to a biological system. These findings suggest that the device can dynamically adjust its memory strength in response to external stimuli, a feature essential for multilevel memory behavior, thereby mimicking the learning behavior observed in the human brain.

A plausible working mechanism for the device was proposed to illustrate the overall memory behavior in the DA-SAM photosynaptic transistors (Figure 5). Upon applying a negative *V*_g_, holes in the p-type transistor were injected into the charge-trapping layer and stabilized by the DA-SAM. Upon light illumination, excitons were generated in the SAM and dissociated into electron–hole pairs. The photogenerated electrons recombined with the trapped holes, while the remaining holes were transported through the HTL into the active layer, resulting in an enhanced photocurrent. Meanwhile, the electron acceptors facilitate the capture and stabilization of excess photoinduced electrons within the SAM layer by lowering the Fermi level of the Py SAM. Despite the comparable LUMO levels of F_4_TCNQ and BCF, the underlying interaction mechanisms with Py differ substantially. In the case of BCF, precoordination with water enables a proton transfer process with Py, rendering the charge-transfer interaction less dependent on precise energy level alignment. This results in a more significant downshift of the Fermi level in the Py SAM, as evidenced by UPS measurements. Consequently, the BCF-based system exhibits improved charge trapping stability compared to F_4_TCNQ. Notably, this effect is particularly prominent in the F_4_BCF system due to its substantial electron deficiency and deep Fermi level, which allows for favorable exciton dissociation and quenching via CT mechanisms, suppresses charge recombination, facilitates stable charge trapping, and maintains a high conductivity state after illumination. Thus, the integration of D–A interactions enables both voltage-induced hole trapping and photoactivated electron storage, achieving bistable memory behavior in a photosynaptic transistor.

**Figure 5:**
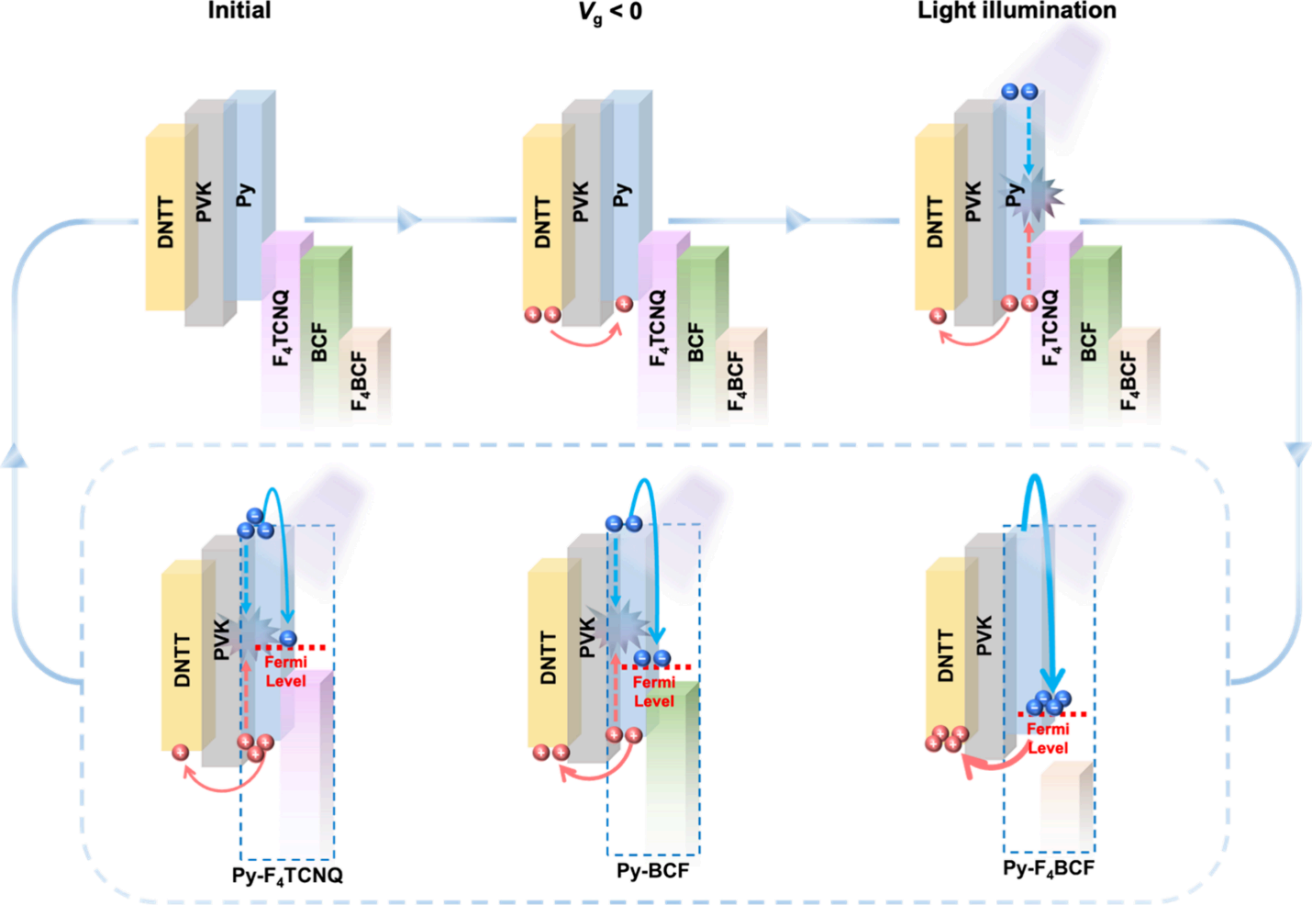
Device working mechanism of the DA-SAM-based photosynaptic transistors: The Lewis-pair F_4_BCF effectively downshifts the SAM’s Fermi level, and with the optimal energy level alignment, promotes the synaptic photoresponse and charge trapping under light illumination. The hole-transporting PVK blocks the hole back-trapping from the channel and electron tunneling from the DA-SAM.

### Image Denoising and Pattern Recognition

Finally, inspired by the human visual system, the integration of optical responsivity and memory functionality in the device makes it highly suitable for neuromorphic computing applications, including advanced image processing. For image denoising tests, 10,000 handwritten digits from the modified National Institute of Standards and Technology (MNIST) data set were corrupted using random noise with a maximum level of 100%. These noisy images were subsequently processed using a denoising array based on the PPF ratios obtained from the F_4_BCF-based synaptic device, in which the signal-to-noise ratio was enhanced, resulting in more apparent digit contours (Figure 6a). This image denoising process was regarded as a preprocessing step. To evaluate its effectiveness, a neural network composed of 784 × 256 × 128 × 10 nodes was employed to recognize the images processed through one, two, and three denoising cycles, as shown in Figure 6b. Since a higher PPF ratio reflects stronger short-term facilitation, higher values lead to more effective noise reduction and clearer restoration of signal features. Remarkably, the Lewis-paired F_4_BCF system, owing to its superior charge stabilization and photoresponse, achieved a high PPF ratio that enabled the recognition accuracy to increase from 56% to 93% after only two denoising cycles, demonstrating excellent denoising capability (Figure 6c). Notably, at an energy consumption of merely 2.96 × 10^–19^ J, the device maintained a PPF ratio of 138%, which was sufficient to perform three cycles of effective image denoising, resulting in a recognition accuracy of ∼90% (Figure S18). These results highlight the potential of the device for energy-efficient neuromorphic preprocessing and pattern recognition tasks. Therefore, the superior performance of the F_4_BCF device in both image processing and information identification underscores the importance of molecular design in tailoring synaptic characteristics for neuromorphic applications.

**Figure 6:**
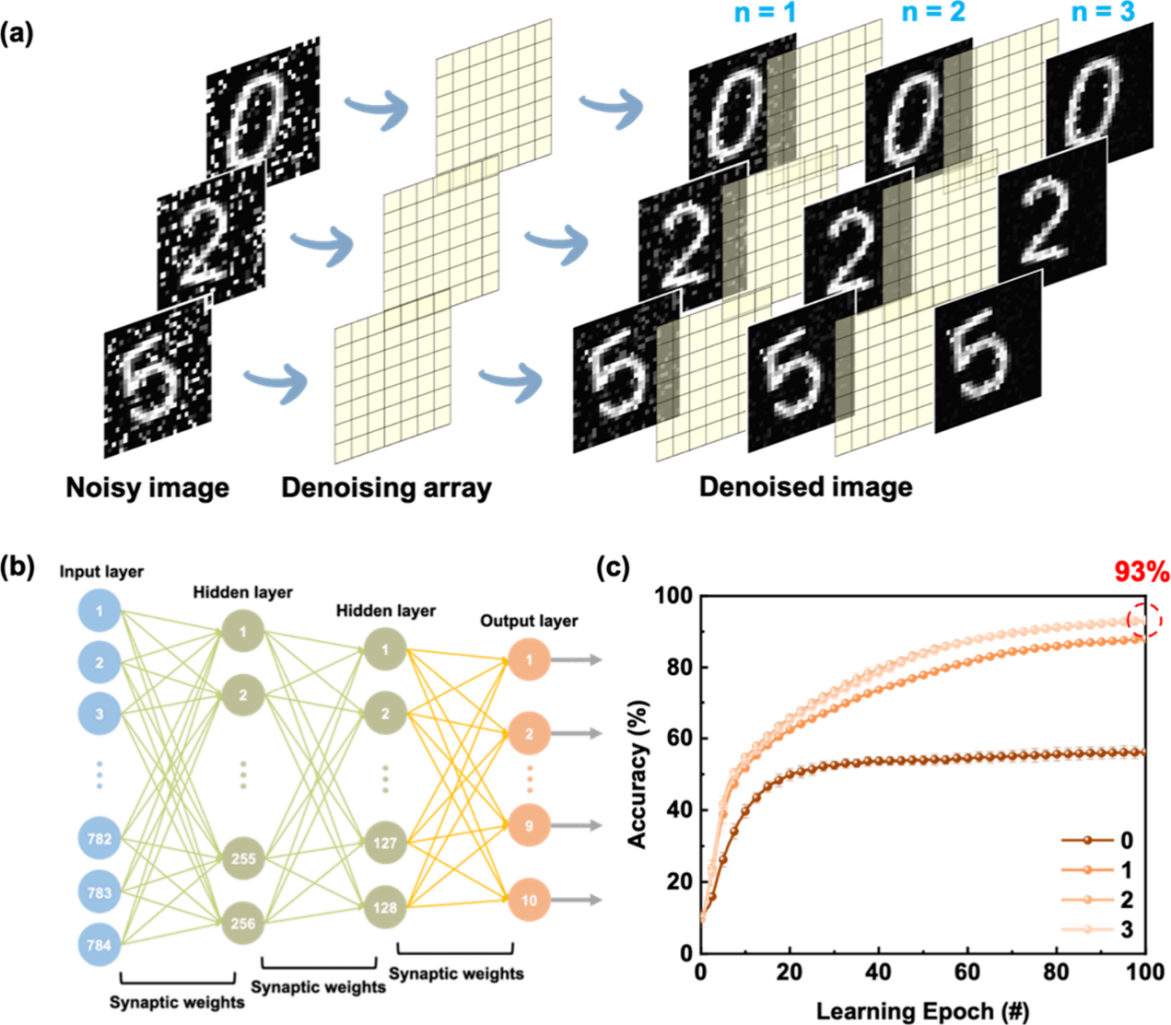
Application demonstration of the photosynaptic transistors with an ultrahigh PPF: (a) Schematic diagram of the denoising process and (b) a neural network architecture with a size of 784 × 256 × 128 × 10. (c) Recognition accuracy for the preprocessed images after different denoising processes and 100 learning epochs.

## Conclusions

In summary, a molecular design strategy that incorporates DA-SAMs into synaptic devices was demonstrated to regulate the Fermi level of the charge-trapping layer effectively. By leveraging the strong electron affinity of the Lewis-paired F_4_BCF and the ultrathin, well-ordered, and densely packed structure of the Py SAM architecture, the CT system facilitates more efficient charge localization and modulation of energy levels. This synergistic effect enhances charge stabilization and improves photoresponse, forming a robust foundation for photosynaptic devices. Notably, this approach results in the following three key achievements: (i) bistable memory behavior via both voltage- and light-induced charge storage, (ii) extraordinary synaptic performance evidenced by the highest reported PPF ratio of 293% and extremely low energy consumption of 2.96 × 10^–19^ J, and (iii) capability in neuromorphic computing, as presented through image denoising and pattern recognition. This achievement is simultaneously the highest PPF and the lowest energy consumption reported in the literature. The results demonstrate that Fermi level tuning through Lewis-paired DA-SAM plays a pivotal role in promoting synaptic characteristics, which holds significant potential for future development of low-power, light-driven neuromorphic devices in vision-based AI systems.

## Supplementary Material



## Notes

Experimental details; water contact angles; optical and photophysical parameters; AFM; ToF-SIMS; electrical characteristics of the photosynaptic transistors (PDF)
