# Supplementary material for: Strategic Fermi Level Engineering of Donor–Acceptor Self-Assembled Monolayer toward Ultrahigh Paired-Pulse Facilitations in Photosynaptic Transistors
Source: J Am Chem Soc. 2025 Sep 15;147(42):38341–53. doi: 10.1021/jacs.5c11235 (PMC12550860; doi:10.1021/jacs.5c11235)
Supplement: Supplementary file 1 [file ja5c11235_si_001.pdf]

Supporting Information

Strategic Fermi Level Engineering of Donor–Acceptor Self-Assembled Monolayer toward Ultrahigh Paired-Pulse Facilitations in Photosynaptic Transistors

Ya-Shuan Wu,[a] Wei-Cheng Chen,[a] Yi-Sa Lin,[b] Cheng-Liang Liu,[c,d] Yan-Cheng Lin[d,e] and Wen-Chang Chen[a,b,d]**

[a] Department of Chemical Engineering, National Taiwan University, Taipei 10617, Taiwan.

[b] Institute of Polymer Science and Engineering, National Taiwan University, Taipei 10607, Taiwan.

[c] Department of Materials Science and Engineering, National Taiwan University, Taipei 10617, Taiwan.

[d] Advanced Research Center of Green Materials Science and Technology, National Taiwan University, Taipei 10617, Taiwan.

[e] Department of Chemical Engineering, National Cheng Kung University, Tainan City 70101, Taiwan.

*Corresponding author. E-mail: ycl@gs.ncku.edu.tw (Y.-C. Lin); chenwc@ntu.edu.tw (W.-C. Chen)

EXPERIMENTAL SECTION

Materials. F_4TCNQ (> 99.0%) was obtained from Ultra Fine Chemical Technology Corp. BCF (> 98.0%) and pyrene (> 97.0%) were purchased from Tokyo Chemical Industry Co., Ltd. Poly(9-vinylcarbazole) (PVK, average M_n = 25,000–50,000) and the anhydrous solvents were supplied by Sigma-Aldrich. All chemicals were used as received without further purification. The synthetic procedures for Py SAM were based on the previously reported work: *ACS Appl. Mater. Interfaces.* **2024**, 16, 69645–69659.

General Characterization. The water contact angles of Py and the DA-SAMs were estimated using CAM110 (Creating Nano Technologies, Inc.). XPS spectra were measured by a Thermo Scientific Nexsa G2. The work functions of Py and the D–A systems were analyzed using UPS (PHI 5000 VersaProbe III, ULVAC-PHI. Inc.). The optimized ground-state geometry of the coordination between Py core and F_4TCNQ was obtained using DFT calculation performed with the Gaussian 09W program, employing the B3LYP method and the 6-31G basis set. EPR was conducted by an EPR-Plus (Bruker) in solution state after 365-nm light illumination for 60 s, and the acceptor concentration was 10 mol%. For optical characterization, a Hitachi U-4100 spectrophotometer was utilized to obtain the UV–vis absorption spectra, and a Jobin Yvon Fluorolog-3 spectrofluorometer with an excitation wavelength (λ_{ex}) of 365 nm was used to provide the PL emission spectra. TR-PL with λ_{ex} = 375 nm was characterized using an optical fiber connected to a spectrometer (iHR 320, Horiba) in NSRRC, Taiwan. The PLQY (λ_{ex} = 368 nm) was estimated using an LED measurement system (LQ-100X, Enlitech). The surface morphology was observed by AFM (Bruker Innova) operating in tapping mode. The device architecture was confirmed by ToF-SIMS analysis (TOF.SIMS 5, IONTOF). The layer thicknesses of the Py and the DA-SAMs were evaluated by an optical thickness meter (OPTM-A3, Otsuka Electronics Co., Ltd.), which are 1.43, 1.57, 1.60, and 1.64 nm for Py, F_4TCNQ, BCF, and F_4BCF systems, respectively.

Fabrication and Characterization of the Photosynaptic Device. First, oxygen plasma-treated SiO_2 (100 nm)/Si substrates were immersed in a 3 mM solution of Py in anhydrous toluene at 70 °C for 6 h under 30–40% humidity to form the Py SAM. After SAM growth, the substrates were rinsed with toluene using an ultrasonic cleaner and dried under vacuum at 80 °C for 15 min to remove the residual solvent. Then, solutions of electron acceptors, including F_4TCNQ, BCF, and F_4BCF, were prepared at a concentration of 10 mM in toluene. Note that for the BCF system, the powder was pre-exposed to the air for 2 h before dissolution to form the Bronsted acid. The Py SAM-modified substrates were then immersed in the respective solutions, F_4TCNQ (60 °C), BCF (room temperature), and F_4BCF (room temperature) for 16 h to allow the formation of CT complexes. After that, the substrates were rinsed with toluene. Post-treatment procedures were performed under the following conditions: F_4TCNQ samples were dried under vacuum at 70 °C for 15 min; BCF samples were kept under ambient conditions for 1 h; and F_4BCF samples were subjected to vacuum drying at room temperature. A PVK layer was spin-coated onto the Py and DA-SAMs at a rate of 4000 rpm for 30 s. For the control device, pyrene and F_4BCF were mixed in a 1:1 molar ratio and spin-coated onto the substrate at 2000 rpm for 60 s. Subsequently, a PVK film was prepared by dissolving PVK at a concentration of 5 mg/mL in THF and spin-coated at 4000 rpm for 30 s. The resulting PVK film was transferred onto the previously spin-coated pyrene–F_4BCF layer. Finally, a 50-nm DNTT was thermally evaporated at a 0.3 Å/s as the semiconducting layer, followed by a 70 nm-thick Au layer deposited at a rate of 0.4–0.5 Å/s as the electrodes using a shadow mask with a channel length (L) and width (W) of 50 and 1000 μm, respectively. Note that the deposition processes were carried out at a pressure of $< 10^{-6}$ Torr.

The memory behavior was conducted using a Keithley 4200-SCS semiconductor parameter analyzer in a nitrogen-filled glovebox at room temperature. The transfer characteristics were measured at $V_d = -50$ V with a V_g ranging from 20 to −60 V. A negative bias ($V_g = -60$ V, 1s) was applied for the electrical writing process, followed by light illumination with a wavelength of 365 nm (3.20 mW/cm^2; 40 s) at $V_d = -50$ V for the photoerasing process and the transient characteristic measurement.

The synaptic performance was confirmed by a Keithley 2634B in a nitrogen-filled glovebox at room temperature. The 365-nm light source was provided by Titan Electro-Optics Co., Ltd., and the light intensity for characterization was 0.40–1.83 mW/cm^2 with a spike width ranging from 50 to 350 ms. The operating V_ds were −1 V for PPF tests and STM–LTM transitions and −0.1 mV for energy consumption measurement. The light intensities were calibrated by a laser power meter (Thorlabs PM 100D), and all measurements were conducted in a dark environment to prevent interference from external light sources.

Image Denoising and Neural Network Simulation. Handwritten digit images were obtained from the MNIST database and used for simulation in MATLAB. To evaluate the denoising performance, random noise was introduced to the original dataset, ranging from 0–100% of the pixel intensity. The brightness values of all images were normalized to a range of 0 to 255. The denoising process utilized a 28 × 28 array composed of PPF ratios derived from an F_4BCF-based device with a spike interval of 50 ms under 365-nm light illumination (0.76 mW/cm^2). Then, the noisy images were repeatedly processed through the denoising array 0–3 times, and the corresponding denoised arrays were converted into gray values, reconstructing the preprocessed digit images. For classification, a neural network with an architecture of 784 × 256 × 128 × 10 was employed, with 80% of the dataset allocated for training and the remaining 20% reserved for testing.

Table S1. Summary of radiative and nonradiative parameters of the D–A systems, including the TR-PL fitting parameters, photoluminescence quantum yields (PLQYs), radiative/nonradiative recombination rates (k_{rad}/k_{nonrad}), charge transfer rate (k_{CT}), and charge transfer efficiency (CTE).

	A_1	τ_1 (ns)	A_2	τ_2 (ns)	τ_{avg} (ns)	PLQY (%)	k_{rad} (ns^{-1})	k_{nonrad} (ns^{-1})	k_{CT} (ns^{-1})	CTE (%)
**Py**	0.64	1.24	0.36	18.44	16.57	56.61	3.4×10^{-2}	0.03	–	–
**F_4TCNQ-0.1**	0.67	1.39	0.33	14.70	12.60	39.16	3.1×10^{-2}	0.05	0.019	24
**F_4TCNQ-0.25**	0.66	1.00	0.34	9.73	8.30	20.14	2.4×10^{-2}	0.10	0.060	50
**F_4TCNQ-0.5**	0.77	0.85	0.23	7.00	5.20	11.69	2.2×10^{-2}	0.17	0.132	69
**BCF-0.1**	0.79	1.08	0.21	6.62	4.53	8.87	2.0×10^{-2}	0.20	0.160	73
**BCF-0.25**	0.83	0.89	0.17	6.33	4.09	1.64	4.0×10^{-3}	0.24	0.184	75
**BCF-0.5**	0.88	0.74	0.12	6.47	3.80	1.18	3.1×10^{-3}	0.26	0.203	77
**F_4BCF-0.1**	0.78	1.03	0.22	8.50	6.29	2.77	4.4×10^{-3}	0.15	0.099	62
**F_4BCF-0.25**	0.91	0.76	0.09	5.16	2.47	1.06	4.3×10^{-3}	0.40	0.345	85
**F_4BCF-0.5**	0.92	0.67	0.08	4.04	1.80	0.33	1.9×10^{-3}	0.55	0.494	89

Table S2. Summary of radiative and nonradiative parameters of the DNTT on different D–A systems, including TR-PL fitting parameters, photoluminescence quantum yields (PLQYs), and radiative/nonradiative recombination rates (k_{rad}/k_{nonrad}).

	A_1	τ_1 (ns)	A_2	τ_2 (ns)	τ_{avg} (ns)	PLQY (%)	k_{rad} (ns^{-1})	k_{nonrad} (ns^{-1})
**DNTT/Py**	0.44	0.15	0.56	0.57	0.49	5.51	1.1×10^{-1}	1.91
**DNTT/F_4TCNQ**	0.46	0.17	0.54	0.60	0.52	4.84	9.3×10^{-2}	1.83
**DNTT/BCF**	0.42	0.16	0.58	0.61	0.53	5.57	1.0×10^{-1}	1.77
**DNTT/F_4BCF**	0.47	0.20	0.53	0.64	0.55	5.30	9.7×10^{-2}	1.73

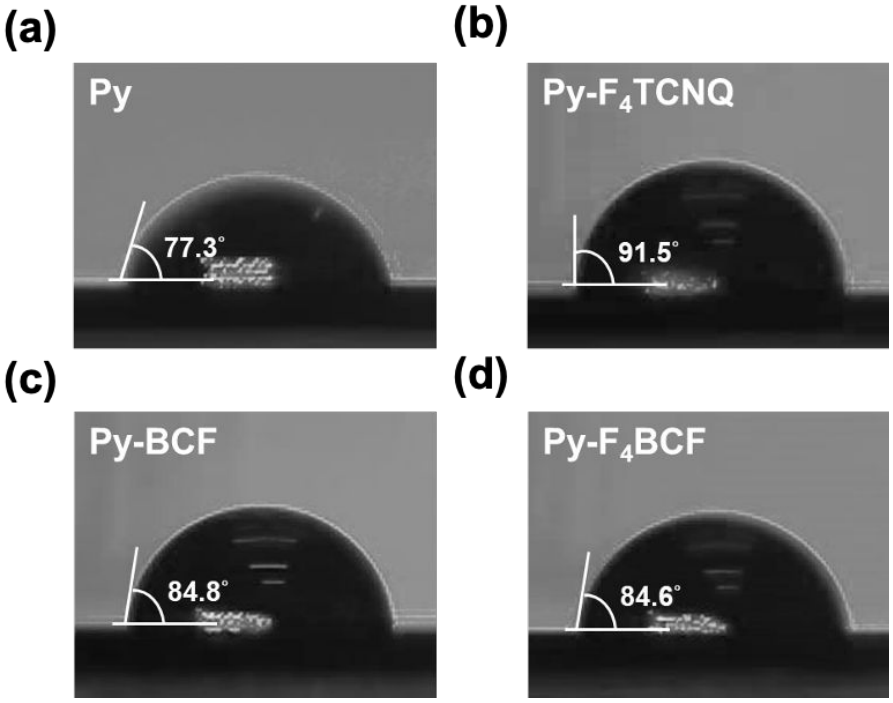

Figure S1. Water contact angles of the self-assembled (a) Py, (b) Py–F4TCNQ, (c) Py–BCF, and d) Py–F4BCF.

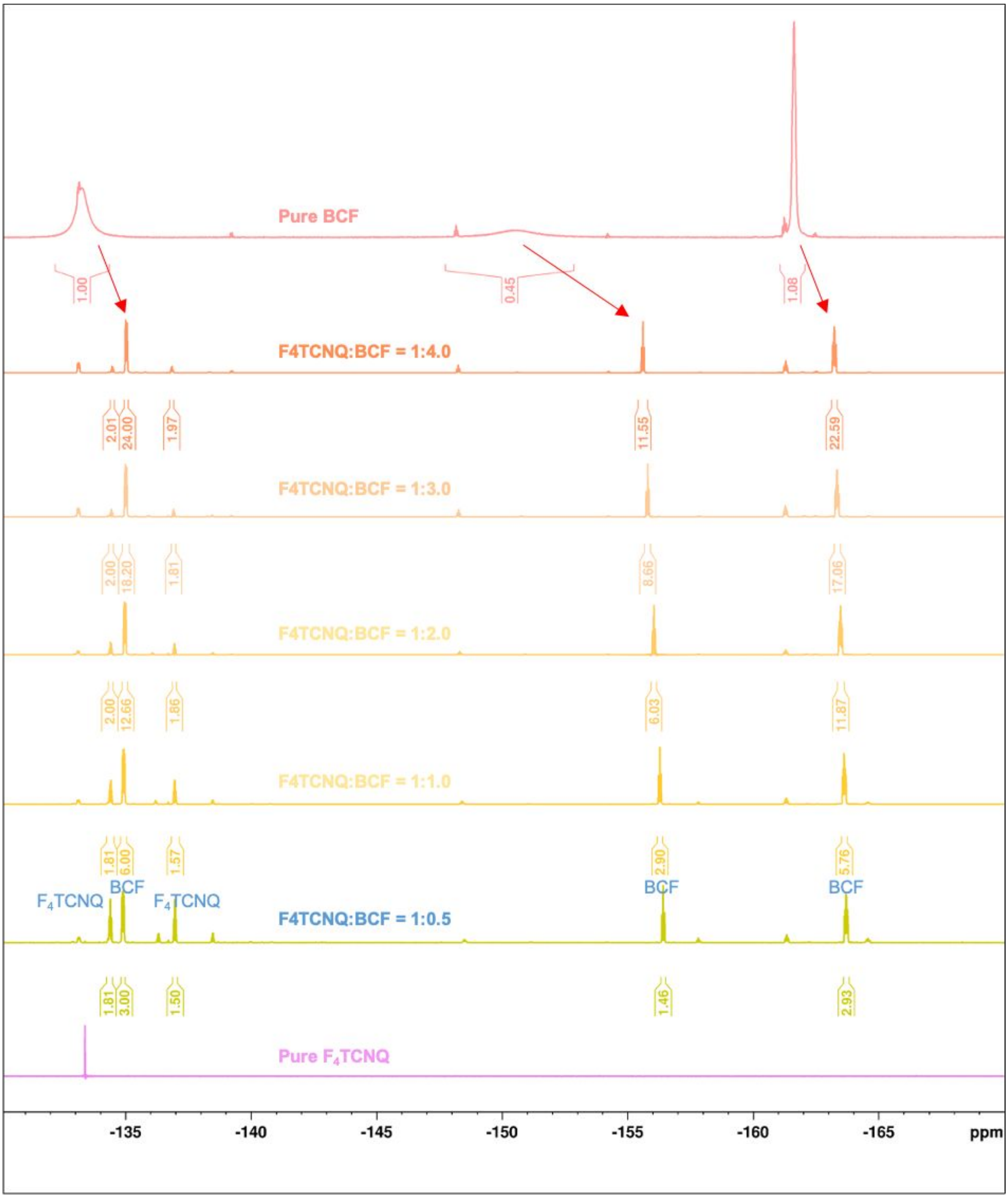

Figure S2. ^{19}F NMR spectra of pure F_4TCNQ, pure BCF, and F_4TCNQ:BCF with varying ratios in toluene-d_8.

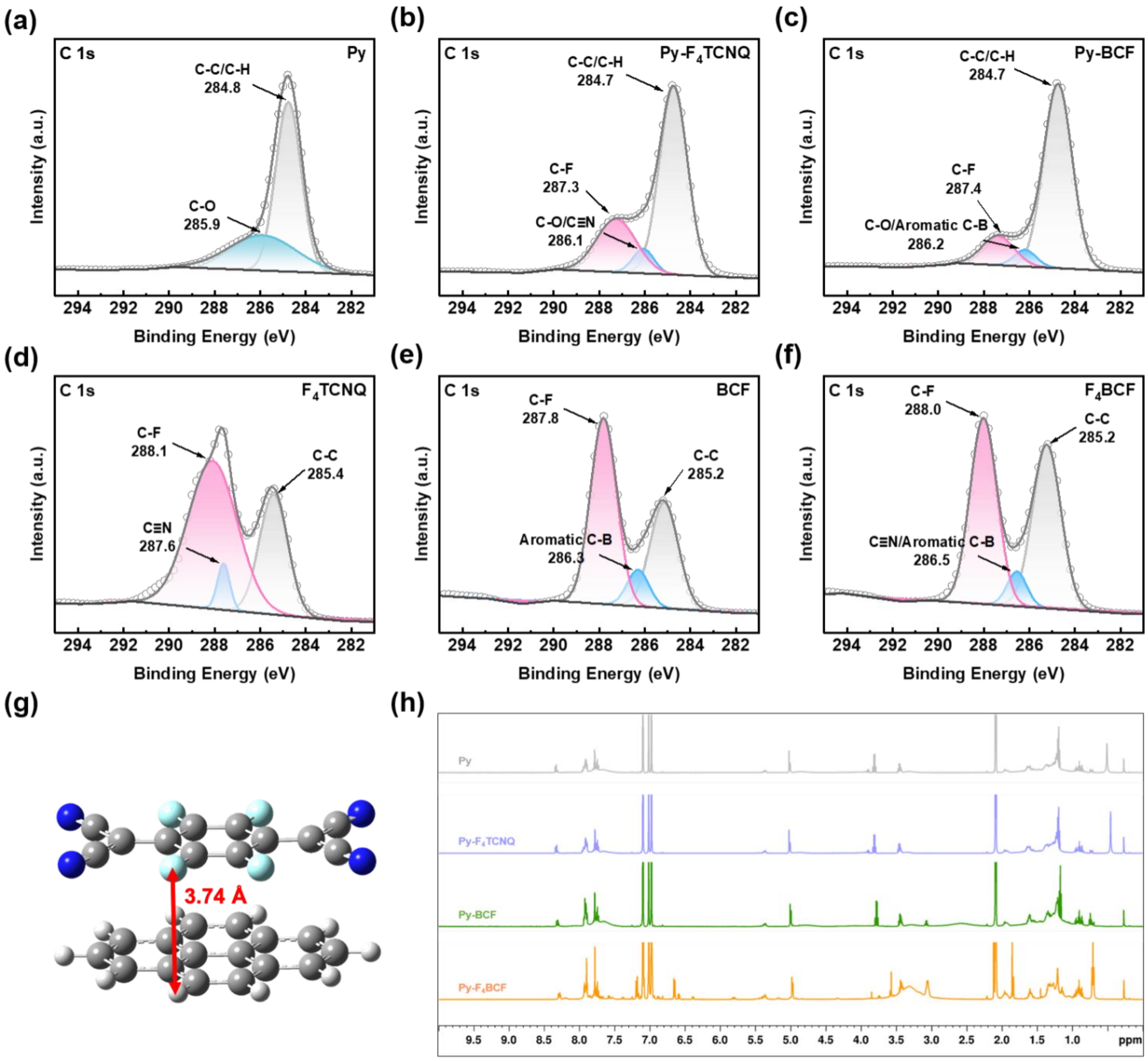

Figure S3. XPS peak deconvolutions of the C-1s band for the self-assembled (a) Py, (b) Py–F_4TCNQ, (c) Py–BCF, (d) F_4TCNQ, (e) BCF, and (f) F_4BCF. (g) DFT calculation of the optimized geometry between the Py core and F_4TCNQ. (h) ^{1}H NMR spectra of Py, Py–F_4TCNQ, Py–BCF, and Py–F_4BCF in toluene-d_8.

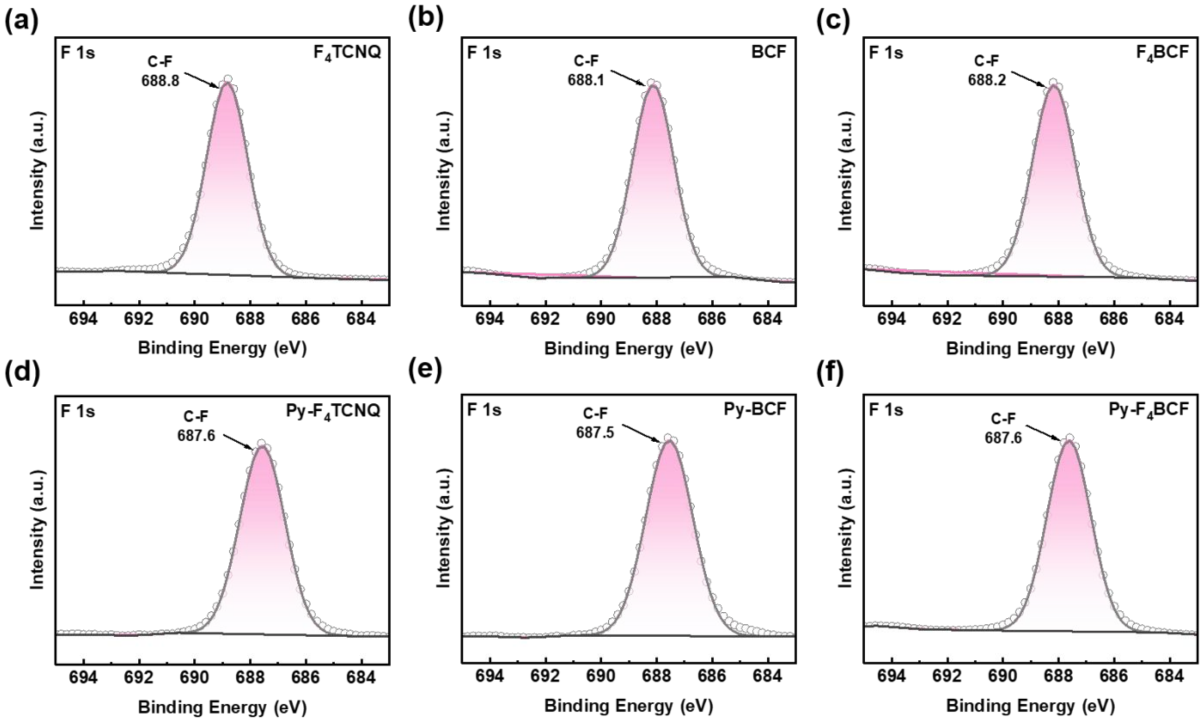

Figure S4. XPS peak deconvolutions of the F-1s band for the Lewis acids of (a) pure F_4TCNQ, (b) pure BCF, and (c) pure F_4BCF, and the self-assembled (d) Py–F_4TCNQ, (e) Py–BCF, and (f) Py–F_4BCF.

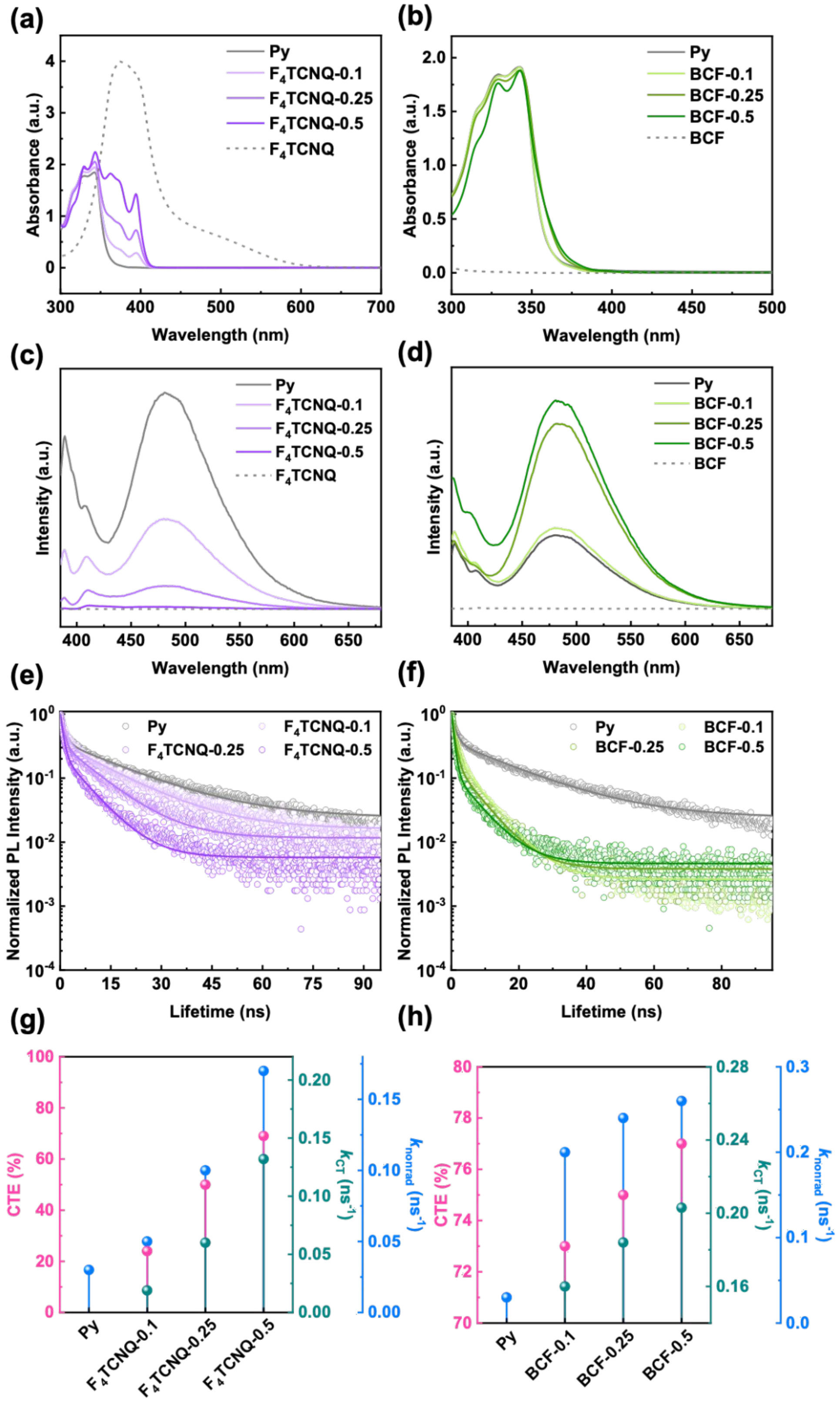

Figure S5. (a,b) UV–vis absorption spectra, (c,d) steady-state PL emission spectra, (e,f) 1-D TR-PL decay profiles, and (g,h) nonradiative parameters including CTE, k_{CT}, and k_{nonrad} of the (a,c,e,g) Py–F_4TCNQ and (b,d,f,h) Py–BCF solutions with an acceptor concentration of 0–50 mol%. Note that the λ_{ex}s are (c,d) 365 nm and (e,f) 375 nm.

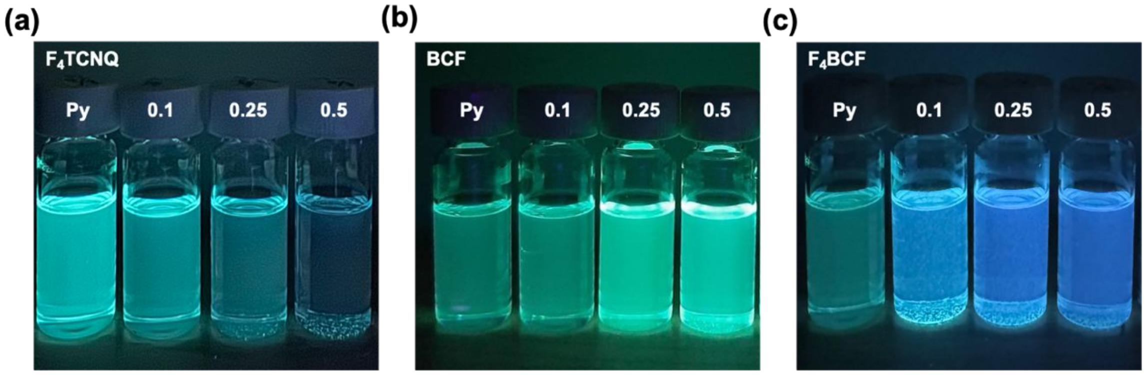

Figure S6. The emission images of (a) Py–F_4TCNQ, (b) Py–BCF, and (c) Py–F_4BCF with an acceptor concentration of 0–50 mol%.

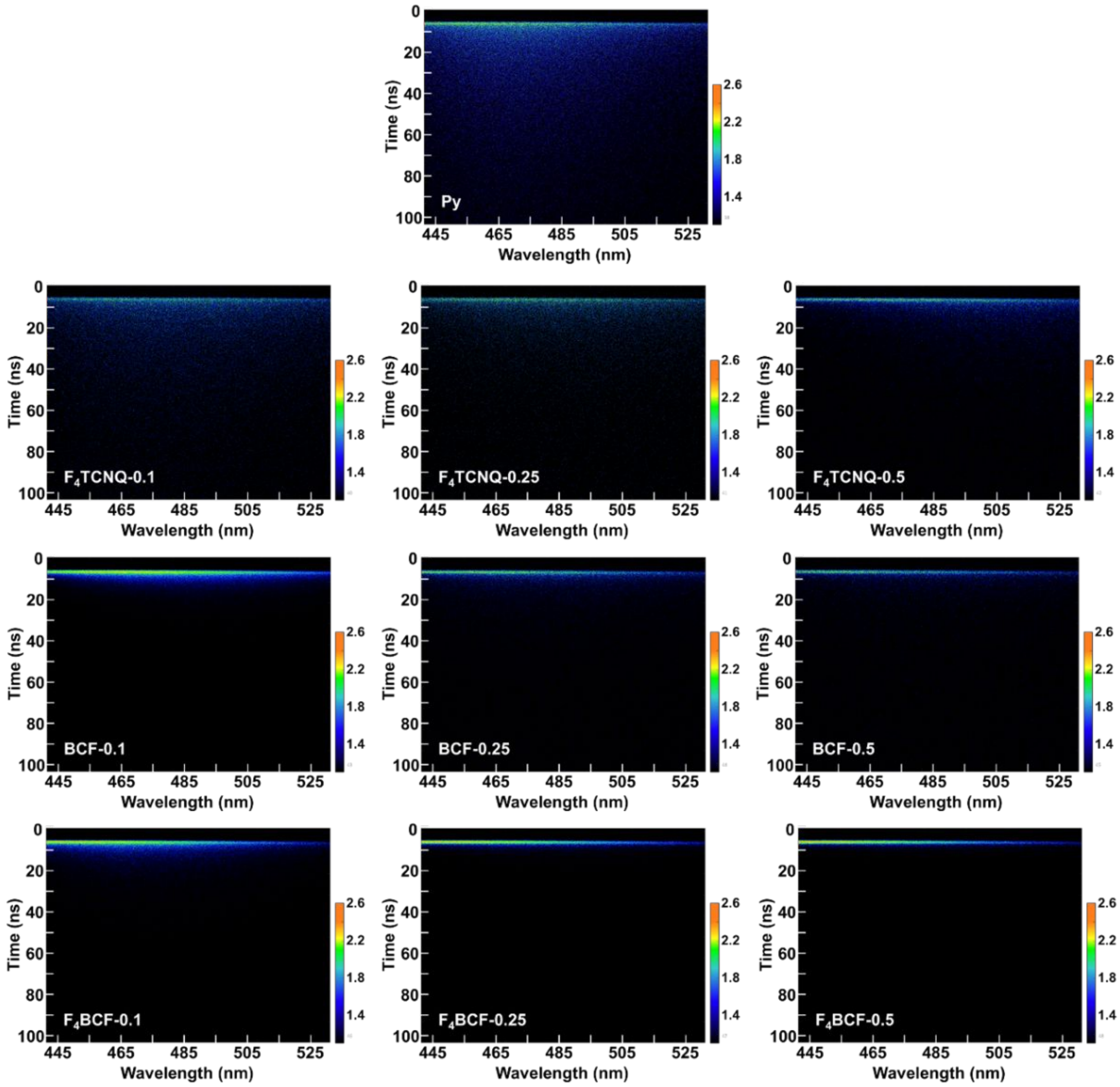

Figure S7. 2-D TR-PL patterns of pure Py, Py–F_4TCNQ (top), Py–BCF (middle), and Py–F_4BCF (bottom) with an acceptor concentration of 0–50 mol%. Note that the λ_{ex} is 375 nm.

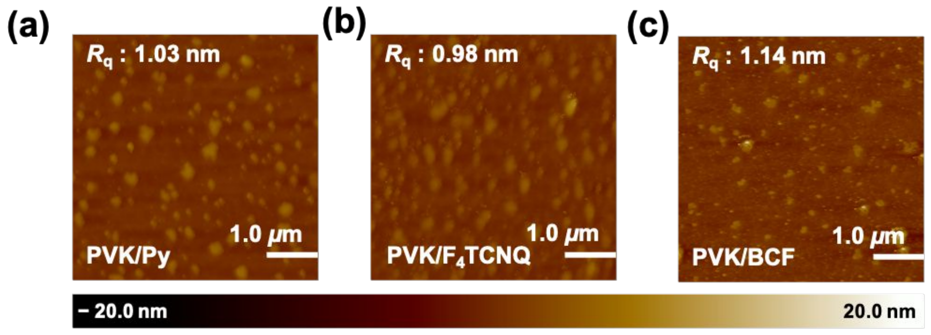

Figure S8. AFM height images of the SAM covered by the hole-transporting layer: (a) PVK/F_4TCNQ, (b) PVK/BCF, and (c) PVK/F_4BCF.

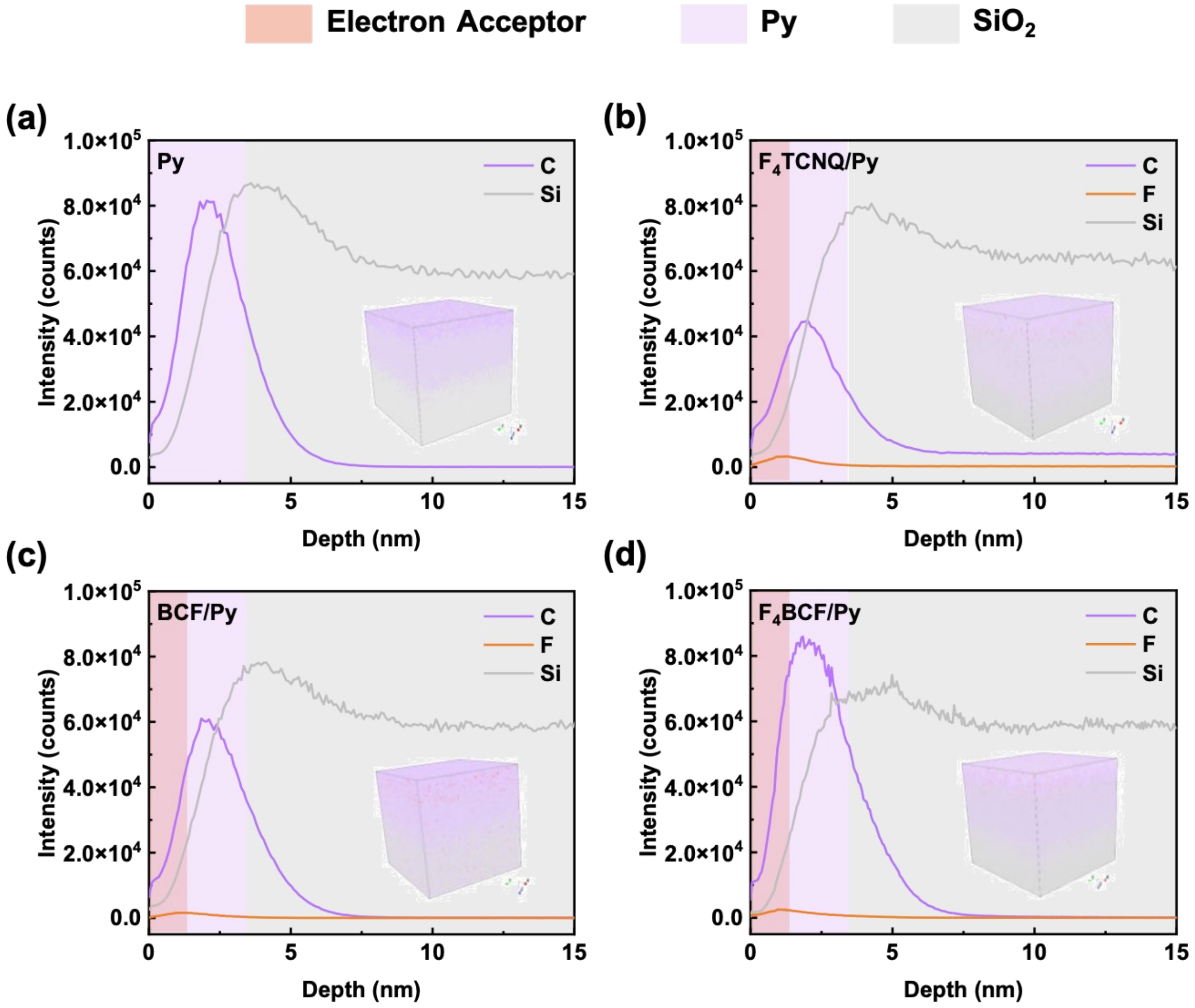

Figure S9. ToF-SIMS 1-D depth profiles and 3-D mappings of the self-assembled (a) Py, (b) F_4TCNQ/Py, (c) BCF/Py, and (d) F_4BCF/Py.

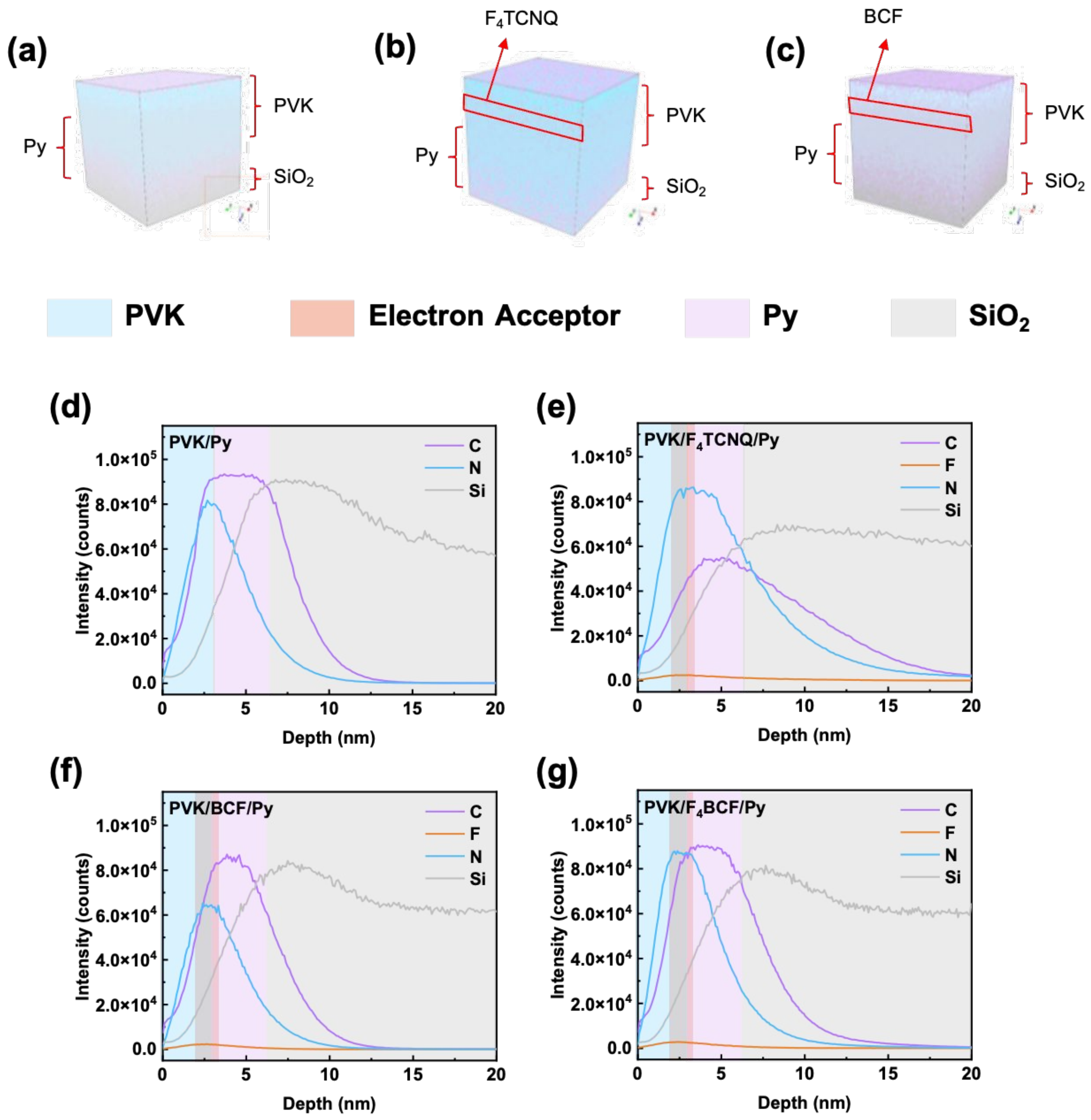

Figure S10. (a–c) ToF-SIMS 3-D mappings and (d–g) 1-D depth profiles of the SAM covered by the hole-transporting layer: (a,d) PVK/Py, (b,e) PVK/F_4TCNQ/Py, (c,f) PVK/BCF/Py, and (g) PVK/F_4BCF/Py.

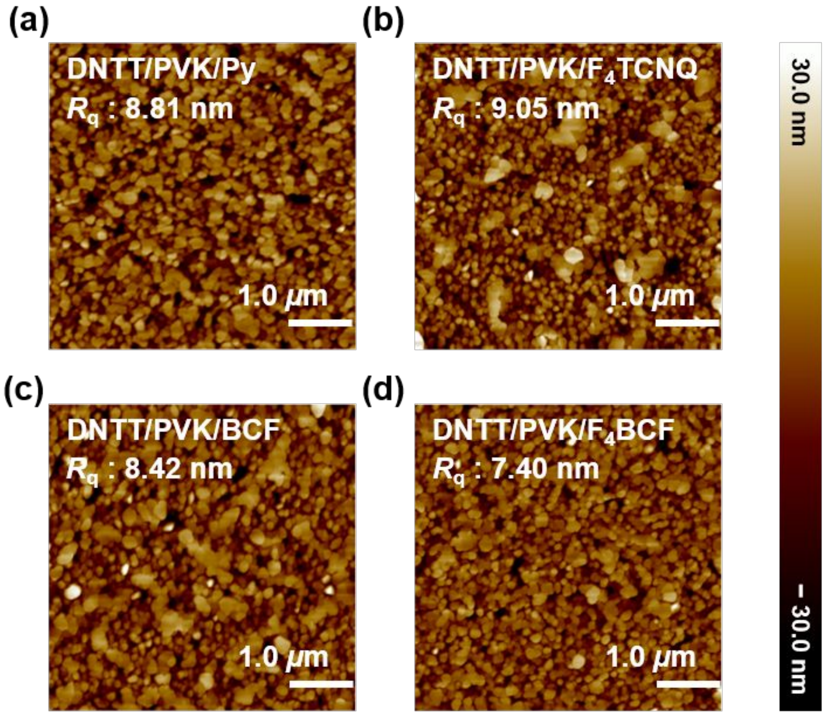

Figure S11. AFM height images of the DNTT thin films deposited on different SAMs: (a) DNTT/PVK/Py, (b) DNTT/PVK/F_4TCNQ, (c) DNTT/PVK/BCF, and (d) DNTT/PVK/F_4BCF.

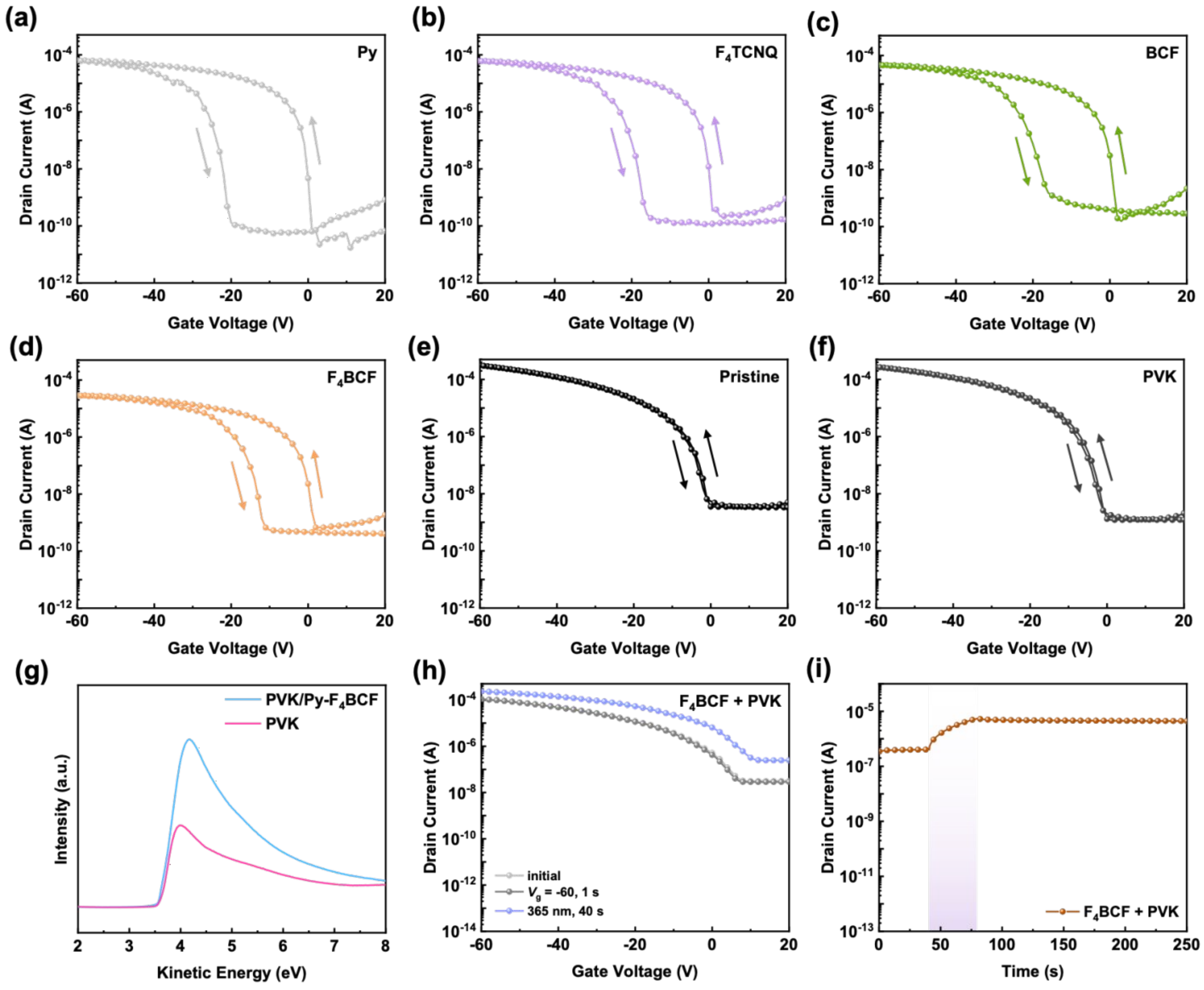

Figure S12. Dual-sweep transfer characteristics of the DNTT deposited onto the self-assembled (a) Py, (b) F_4TCNQ/Py, (c) BCF/Py, and (d) F_4BCF/Py. The reference devices with DNTT deposited onto the (e) SiO_2 surface and (f) PVK layer. (g) UPS spectra of the pristine PVK and PVK/Py-F_4BCF. (h) Transfer characteristics of the device with DNTT deposited onto PVK film, blending with F_4BCF. The measurements were conducted at $V_d = -50$ V with the V_g forward sweeping from 20 to –60 V. (i) Transient characteristics of the device with DNTT deposited onto PVK film blending with F_4BCF under 365 nm-light illumination for 40 s at $V_d = -50$ V.

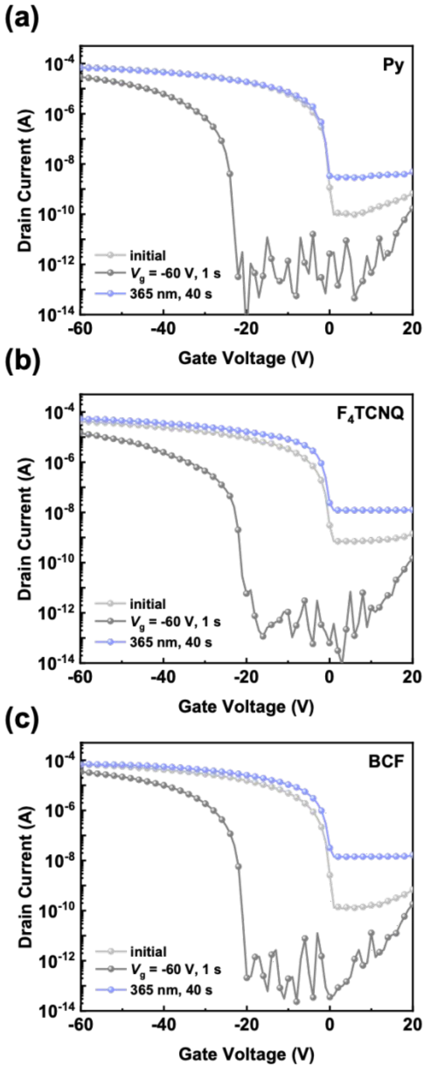

Figure S13. Transfer characteristics of the devices with DNTT deposited onto the self-assembled (a) Py, (b) F_4TCNQ/Py, and (c) BCF/Py under the illumination of 365-nm light (3.20 mW/cm^2) at V_d = −50 V and with the V_g forward sweeping from 20 to –60 V.

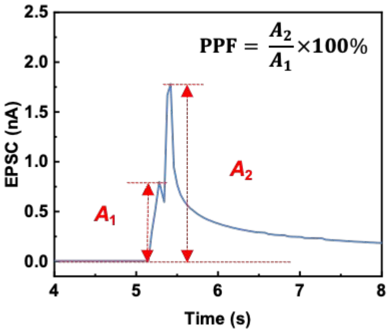

Figure S14. Illustration of the PPF ratio calculation.

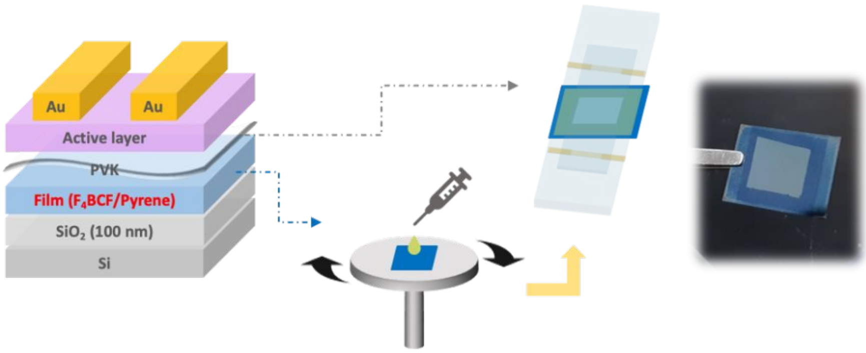

Figure S15. Schematic diagram of the device fabrication process for the spin-coated control device with bilayered thin films of F_4BCF/Py and PVK. Note that the PVK layer was transferred from a soft elastomer due to the overlapped solubility between PVK and the components in F_4BCF/Py.

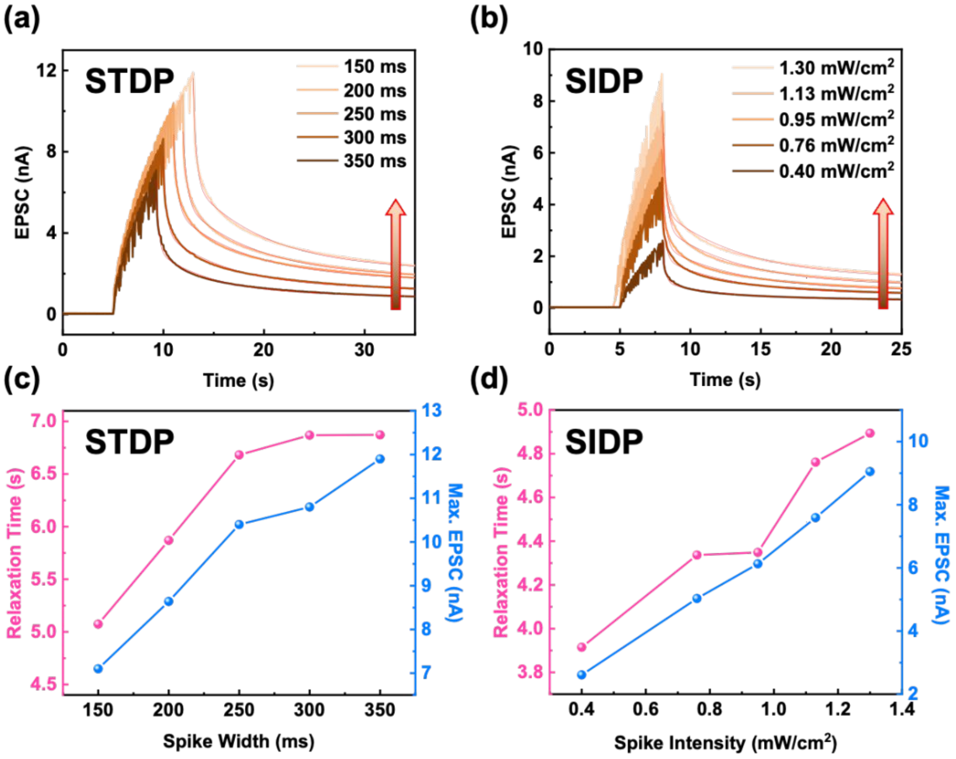

Figure S16. Photosynaptic transistor characteristics: (a,b) STM–LTM transitions and (c,d) the relationship between relaxation times and maximum EPSCs for (a,c) STDP and (b,d) SIDP based on the F_4BCF device by tuning the spike widths and light intensities. Note that the device was measured under 365 nm-light illumination at $V_d = -1$ V.

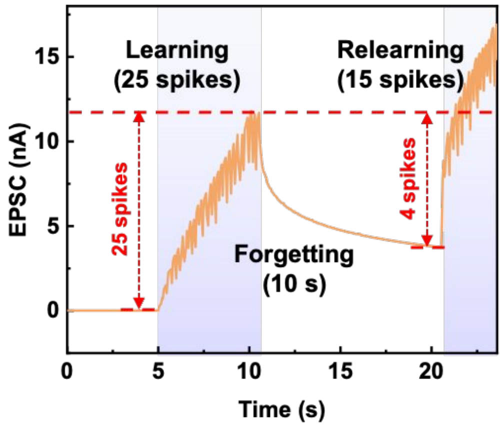

Figure S17. Photosynaptic transistor characteristics: Emulation of the learning–forgetting–relearning behavior in the human brain based on the F_4BCF device with 365-nm light (0.76 mW/cm^2; spike width = 100 ms) illumination at $V_d = -1$ V.

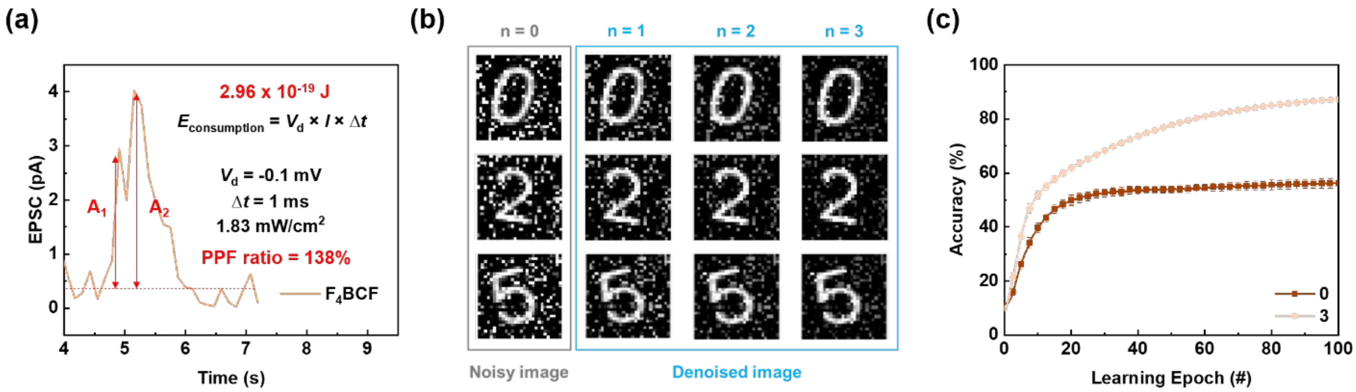

Figure S18. (a) Calculation of PPF ratio under ultralow energy consumption. (b) Noisy input images (n = 0) and denoised results after 1–3 synaptic processing steps. (c) The recognition accuracy for the preprocessed images after different denoising processes and 100 learning epochs.
